# Identifying important questions for Cochrane systematic reviews in Eyes and Vision: Report of a priority setting exercise

**DOI:** 10.1002/cesm.12014

**Published:** 2023-05-24

**Authors:** Jennifer R. Evans, Iris Gordon, Augusto Azuara‐Blanco, Michael Bowen, Tasanee Braithwaite, Roxanne Crosby‐Nwaobi, Stephen Gichuhi, Ruth E. Hogg, Tianjing Li, Virginia Minogue, Roses Parker, Fiona J. Rowe, Anupa Shah, Gianni Virgili, Jacqueline Ramke, John G. Lawrenson, Cochrane Eyes and Vision Priority Setting Group Authorship

**Affiliations:** ^1^ Centre for Public Health, School of Medicine, Dentistry and Biomedical Sciences Queen's University Belfast Belfast UK; ^2^ International Centre for Eye Health, Department of Clinical Research London School of Hygiene & Tropical Medicine London UK; ^3^ Knowledge & Research Department College of Optometrists London UK; ^4^ School of Population and Life Course Sciences and School of Immunology and Microbial Sciences King's College London London UK; ^5^ The Medical Eye Unit Guy's and St Thomas' NHS Foundation Trust London UK; ^6^ Research and Development Department Moorfields Eye Hospital and Institute of Ophthalmology, UCL London UK; ^7^ Department of Ophthalmology University of Nairobi Nairobi Kenya; ^8^ Department of Ophthalmology, School of Medicine University of Colorado Anschutz Medical Campus Denver Colorado USA; ^9^ Member of Cochrane Consumer Network London UK; ^10^ The Cochrane Collaboration London UK; ^11^ Institute of Population Health University of Liverpool Liverpool UK; ^12^ School of Optometry and Vision Science University of Auckland Auckland New Zealand; ^13^ School of Health and Psychological Sciences, Centre for Applied Vision Research, City University of London London UK

**Keywords:** equity, Eyes and Vision, prioritization, systematic reviews

## Abstract

**Introduction:**

Systematic reviews are important to inform decision‐making for evidence‐based health care and patient choice. Deciding which reviews should be prioritized is a key issue for decision‐makers and researchers. Cochrane Eyes and Vision conducted a priority setting exercise for systematic reviews in eye health care.

**Methods:**

We established a steering group including practitioners, patient organizations, and researchers. To identify potential systematic review questions, we searched global policy reports, research prioritization exercises, guidelines, systematic review databases, and the Cochrane Library (CENTRAL). We grouped questions into separate condition lists and conducted a two‐round online modified Delphi survey, including a ranking request. Participants in the survey were recruited through social media and the networks of the steering group.

**Results:**

In Round 1, 343 people ranked one or more of the condition lists. Participants were eye care practitioners (69%), researchers (37%), patients or carers (24%), research providers/funders (5%), or noneye health care practitioners (4%) and from all World Health Organization regions. Two hundred twenty‐six people expressed interest in completing Round 2 and 160 of these (71%) completed the Round 2 survey. Reviews on cataract and refractive error, reviews relevant to children, and reviews on rehabilitation were considered to have an important impact on the magnitude of disease and equity. Narrative comments emphasized the need for reviews on access to eye health care, particularly for underserved groups, including people with intellectual disabilities.

**Conclusion:**

A global group of stakeholders prioritized questions on the effective and equitable delivery of services for eye health care. When considering the impact of systematic reviews in terms of reducing the burden of eye conditions, equity is clearly an important criterion to consider in priority‐setting exercises.

## 1 INTRODUCTION

Cochrane Eyes and Vision is an international network of individuals working to prepare, maintain and promote access to systematic reviews of interventions to treat, prevent, or diagnose eye conditions or vision impairment [1]. The intended beneficiaries of the work of Cochrane Eyes and Vision are people making health care decisions for eye health care. Eyes and Vision includes conditions that are common and have a high global disease burden, for example, cataract and dry eye disease, and conditions that are rare but have a high impact on quality of life and high individual cost such as eye cancer.

Systematic reviews aim to use explicit and structured methods to minimize bias in the selection and analysis of studies, and, by combining the results of different studies, estimate treatment effects and other parameters with greater precision [2]. Over the last 30 years, there have been substantial methodological developments in the science of evidence synthesis. Building on the methods for systematic reviews of interventions [3], there are now many different types of evidence synthesis including reviews of diagnostic test accuracy studies [4], prognostic factor studies [5], and methods for mapping out the literature (scoping reviews) [6]. It is important to consider “equity,” that is, the effect of health interventions on underserved populations and to provide evidence to support the reduction in the health disparities for the social axes along which they occur in different settings, including gender, socioeconomic status, and social support. Over the last decade, formal assessment of equity and methods to assess disparities in care in Cochrane systematic reviews have been developed [7]. Cochrane Eyes and Vision has begun to apply an equity lens to the group's reviews [8] and focus on supporting global efforts for Universal Health Care in Eyes and Vision [9]. Given the crucial role of systematic reviews in informing evidence‐based health care provision and decision‐making, deciding which reviews should be prioritized is a key issue.

Cochrane Eyes and Vision has published 247 systematic reviews (to end of 2022) including reviews of interventions and diagnostic test accuracy [10]. More recently, the group has supported reviews of prognostic factors [11]. Cochrane Eyes and Vision reviews primarily address questions relevant to clinical research (treatment, diagnosis, prognosis) but also potentially cover public health, health services, and implementation research. In response both to funding changes and reflecting on the experience of producing rapid evidence syntheses in the COVID era, Cochrane is building a new model for developing and publishing systematic reviews [12]. In the future, community engagement with stakeholders, including priority setting and dissemination of the results of reviews, will be taken on by thematic hubs. Since registration as a group in 1997, Cochrane Eyes and Vision has prioritized reviews by responding to review author requests and working with other stakeholders such as guideline panels [13, 14, 15, 16, 17, 18] and the World Health Organization (WHO); members of the group were also involved in the James Lind Alliance process Sight Loss and Vision Priority Setting Partnership [19] and the more recent Grand Challenges in Global Eye Health prioritization exercise [20]. The purpose of the current priority setting process was to reach a broader group of stakeholders than these previous exercises and assess systematically the questions relevant to our scope.

## 2 AIM

The Cochrane Eyes and Vision Priority Setting Exercise aimed to generate and publicize a list of priority topics, for both new and updated reviews, ensuring the involvement of our main stakeholders.

## 3 METHODS

The protocol for this work has previously been published [21]. This project was approved by the ethics committee of the London School of Hygiene & Tropical Medicine (22669, December 2020).

In brief, we established a steering group including ophthalmologists, optometrists, orthoptists, ophthalmic nurses and relevant professional bodies, patient organizations, experienced clinical editors, systematic review methodologists, and information specialists by drawing on the collaborative networks of Cochrane Eyes and Vision. Members of this group had been involved in previous priority setting exercises [19, 20].

We identified and summarized potential priority questions by scrutinizing the following sources. Searches were done by an experienced information specialist and are listed in Supporting Information: Appendix 3.

- Global policy reports
- Other research prioritization and roadmaps
- High‐quality guidelines identified during the course of a collaborative project with WHO [9]
- Cochrane Eyes and Vision systematic reviews
- Cochrane Eyes and Vision US Project Database of Systematic Reviews in Eyes and Vision [22]
- Intervention studies on the Cochrane Library (CENTRAL)

Using this information, we prepared a list of potential review topics for each of 13 condition groups: cataract, refractive error, diabetic retinopathy, glaucoma, macular disease, other retinal conditions, ocular surface disease, eyelid and lacrimal system, infection and inflammation, neuro‐ophthalmology, children, rehabilitation, and general. Questions were removed where:

- high quality, current systematic reviews exist, and new trials are either unlikely to have been done, or if they have been done, are unlikely to change the conclusions of the review; we largely based this on our knowledge of Cochrane reviews recently published or in progress.
- the topic was considered out of scope because either it does not address a condition of interest, or it does not address a relevant clinical question;
- the question was unclear or ill‐defined or cannot be answered by either intervention, diagnostic test accuracy, prognostic, or scoping review.

With the assistance of the Steering Group and Cochrane Eyes and Vision editors, these lists were reviewed and grouped as necessary to obtain 10 questions for each topic group.

We elicited the input of stakeholders using a two‐stage modified Delphi method via an online survey in Qualtrics (Qualtrics). The survey was pilot‐tested by steering group members and Cochrane Eyes and Vision editors. We reached out to stakeholders using social media and circulating within Cochrane and steering group networks. There were no restrictions on who could contribute to the survey. In Round 1, for each condition group, participants were presented with 10 questions and asked to choose the top five review questions in order of importance by adding a number in the box next to the question: 5 = *most important* to 1 = *least important of the top five selected reviews*. For each condition, there was also the opportunity to provide information on additional review questions, and/or additional comments, in a free text box. Average scores for each question were used to rank the 10 questions for each condition group. In Round 2, participants were presented with the top five questions ranked in Round 1. New review questions were collated, and any question falling within scope and identified by more than two participants was added to the Round 2 list. In Round 2 for each question, the percentage of responses definitely/possibly yes were tabulated for the following four importance criteria. These were criteria developed and used by the Cochrane Eyes and Vision editorial team when considering the choice of new review topics, formalized here for the purpose of the online survey.

- does the proposed new review (or review update) address an important uncertainty?
- will a new review (or review update) at this point in time resolve this uncertainty?
- to what extent would resolving this uncertainty reduce the magnitude of vision impairment and eye health disorders?
- to what extent would resolving this uncertainty reduce inequalities/disparities in (i.e., have an equalizing effect of) the magnitude of disease or access to care for vision impairment or eye health disorders?

No reimbursement for participation was offered apart from acknowledgment under group authorship for participation in both rounds of the survey. Results of the survey were fed back to participants by email and via the Cochrane Eyes and Vision website. Feedback on the process was elicited by survey using the following two questions:

a. What did stakeholders like about the process?
b. What did stakeholders want to improve about the process?

## 4 RESULTS

Searches were done over the period January to June 2021. We opened the Round 1 survey on December 16, 2021, and closed on March 31, 2022. Round 2 was open to participants who registered an interest between May 1 and June 1, 2022. A total of 343 participants completed Round 1 and 226 of these expressed interest in taking part in Round 2; 160 (71%) of these participants went on to complete Round 2 (Table 1).

**Table 1:** Characteristics of respondents Round 1 and Round 2.

*N* (%)	Round 1 (*n* = 343)	Round 2 (*n* = 160)
Gender		
Male	142 (41)	80 (50)
Female	198 (58)	79 (49)
Prefer not to say	3	1
WHO region		
African region	38 (11)	20 (13)
Region of the Americas	19 (6)	13 (8)
Eastern Mediterranean region	10 (3)	8 (5)
European region	223 (65)	84 (53)
South East Asian region	17 (5)	13 (8)
Western Pacific region	33 (10)	21 (13)
Missing	3	1
Status^a^		
Patient or carer	82 (24)	21 (13)
Practitioner—eye care	237 (69)	123 (77)^b^
Practitioner—other	12 (4)	10 (6)
Researcher	126 (37)	86 (54)
Provider	18 (5)	10 (6)
Other	1	3 (2)

Abbreviation: WHO, World Health Organization.

^a^ More than one category may apply.

^b^ Ophthalmologist (77), optometrist (34), orthoptist (6), nurse (1), other (5).

Questions presented and ranked in the online survey in Round 1 and Round 2 are listed in Appendix 4 Supporting Information: Table S1. Scores for importance criteria are presented in Appendix 4 Supporting Information: Table S2. Additional questions proposed by participants are summarized in Appendix 4 Supporting Information: Table S3.

Additional comments from respondents emphasized the difficulty of making choices with such diverse topics “Tough decision! They are all important!.” There was also an issue with technical language: some of the respondents who were not involved in research, or were not practitioners, did not understand what some of the topics meant. “Eye research needs to address patient needs and benefits, as expressed by them, not in technical language.”

Equity was an important issue for respondents: a general comment that was made for several conditions was “As a note, for the topics selected it would be great to have disaggregated data from studies included to review gender, socioeconomic and other disparities that may exist in each question area.” Another participant noted that “All of the above tend to focus on the cognitively able population, it would be helpful to see research that addresses the needs of those less cognitively able (dementia, intellectual disabilities) to see what interventions in all the highlighted cases may work. There is a lack of a clinically validated functional vision assessment tool that can be used with these populations but would be incredibly helpful to have.”

Some of the additional questions suggested by participants mapped or were related to review questions in the top 10 list already. We have included these questions in separate tables to inform potential review teams of the likely scope of the question/issues to consider (Supporting Information: Table S3). Some questions and comments, although representing important potential reviews and research questions, were out of scope for Cochrane Eyes and Vision Reviews, such as reviews of prevalence; reviews of the coverage and quality of services around the world or review of specific aspects of a health system; and review questions on conditions covered by other Cochrane Review Groups (e.g., retinopathy of prematurity is covered by Cochrane Neonatal and interventions for the management of headache in idiopathic intracranial hypertension covered by Cochrane Pain, Palliative and Supportive Care) (Supporting Information: Table S4). In addition, some suggested review questions were already covered by reasonably up‐to‐date Cochrane Reviews or review updates in progress [23, 24, 25, 26, 27, 28] or were useful pointers to important outcomes to be considered in reviews, in particular impact on social, economic, educational, and quality of life outcomes and cost‐effectiveness of interventions (Box 1).

Box 1 **Summarizes the condition‐specific rankings**

**Table:** 

*Cataract*
Participants considering priority questions for cataract emphasized access and affordability, training for eye care practitioners, and monitoring of outcomes (Supporting Information: Table S1). For the top prioritized review question for cataract “Interventions to improve access to, and/or affordability of, cataract surgery” over 90% of respondents felt this was an important uncertainty and reducing this uncertainty would have an important impact on the magnitude of eye disease and equity (Supporting Information: Table S2). Most of the additional questions suggested for cataract in the first round mapped well to the top 10 list (Supporting Information: Table S3).
*Refractive error*
Questions prioritized for refractive error included questions on uptake, access, and practitioner training including prescribing guidelines (Supporting Information: Table S1). Questions relating to the development and progression of refractive error and interventions for presbyopia were in the top five questions. For the top question, “Models to increase uptake and access to refraction and optical services” over 90% of respondents felt that this question addressed all four importance criteria (Supporting Information: Table S2). A number of additional questions related to this topic were suggested and are included for information in Supporting Information: Table S3.
*Diabetic retinopathy*
Respondents prioritized review questions on the integration of diabetes eye care services within the health system and overcoming barriers/increasing attendance for eye checks and screening for people with diabetes, including the role of artificial intelligence (Supporting Information: Table S1). Noninvasive treatments for slowing down progression of diabetic retinopathy were also seen as important—this question was identified from the 2014 James Lind Alliance Priority Setting Partnership in Sight Loss and Vision. Questions related to increasing attendance and overcoming barriers were judged to have the most impact on equity; noninvasive treatments less so (Supporting Information: Table S2).
*Glaucoma*
Questions related to monitoring glaucoma and improving the referral pathway, including telemetry (remote monitoring of patients), artificial intelligence, and optical coherence tomography, were ranked highest (Supporting Information: Table S1). Alongside this, a review on prognostic factors for glaucoma development and progression was prioritized. The first prioritized question “Monitoring glaucoma to prevent progression, including with telemetry” was judged to have the most impact on the four important criteria (Supporting Information: Table S2).
*Macular disease and other retinal conditions*
Similar to glaucoma, reviews relating to treatment pathways and monitoring (with telemetry) were considered high priority (Supporting Information: Table S1). Several questions relating to prognosis were also rated: one on the progression of age‐related macular degeneration (AMD), “Prognostic factors for the development and progression of AMD” one for the prognosis of inherited retinal diseases “Prognostic factors for visual loss in inherited retinal diseases” and for the outcome of surgery “Prognostic factors for the outcome of surgery for epiretinal membranes.” The safety and efficacy of different drug delivery systems for the treatment of AMD was considered a priority, as was “Educational interventions for wellbeing/quality of life for people with AMD.” Detection of retinal detachment in noneye care settings was also prioritized. In general, reviews on macular disease and other retinal conditions were rated less highly on the importance criteria with none of the top five prioritized questions scoring over 90%.
*Ocular surface disease and eyelid*
Reviews on ocular surface disease and the eyelid and lacrimal system were scored lower against the importance criteria (Supporting Information: Table S2) and included reviews on the diagnosis and treatment of dry eye, treatments for blepharitis and meibomian gland dysfunction, ptosis, lacrimal duct obstruction, entropion, and cancers involving the eyelid (Supporting Information: Table S1). Other review questions included “Interventions to improve the outcomes of corneal transplantation” and “Prognostic factors for development of dry eye syndrome, including biomarkers.” One additional comment emphasized the importance of having regular updates on the growing number of interventions for dry eye. This would seem to be a key area and experience with developing a network meta‐analysis and living systematic review for interventions to control myopia may be relevant to this topic area where there are a number of individual Cochrane Reviews available already, some of which may need to be updated.
*Neuro‐ophthalmology*
Intervention reviews for optic neuritis not associated with multiple sclerosis and for idiopathic intracranial hypertension were prioritized alongside two prognosis reviews for visual field loss in acquired brain injury and vision loss in intracranial hypertension (Supporting Information: Table S1). These topics were given a moderate importance criteria rating (Supporting Information: Table S2). Several respondents felt that the management of persisting headache in idiopathic intracranial hypertension (i.e., headache following effective intervention to normalize cerebrospinal fluid pressure) should be prioritized. The management of pain (rather than vision problems or headache resulting from high cerebrospinal fluid pressure) is more appropriately covered by Cochrane Pain, Palliative, and Supportive Care with expertize in pain. Diagnosis and gestational management in idiopathic intracranial hypertension and idiopathic intracranial hypertension without papilloedema were also flagged up.
*Infection and inflammation*
Prevention of corneal infections in contact lens wearers was prioritized. Reviews on interventions for both infectious and noninfectious uveitis as well as prognostic factors for the development and progression of uveitis were also given priority (Supporting Information: Table S1). Interventions for the prevention and treatment of recurrent ophthalmic herpes were similarly highlighted; the Cochrane Review for the latter was last published in 2015 and this ranking indicates that it should be a priority for updating.
*Children*
A review of school vision screening programs (methods and impact) scored highly on the ranking and importance criteria (Supporting Information: Tables S1 and S2). Two reviews of screening (amblyopia, retinoblastoma) and treatments for amblyopia were highlighted. One respondent suggested “Guidelines for managing refractive error, strabismus and amblyopia in low resource settings,” but it was felt that guidelines were not a suitable topic for systematic reviews and should be managed in the local context.
*Rehabilitation*
Rehabilitation review questions scored well including “Interventions to improve access and affordability of visual rehabilitation” and “Models of rehabilitation including integration with eye care” (Supporting Information: Tables S1 and S2). Assistive technology for adults and children with low vision and psychological interventions to support living with visual impairment were in the top five reviews as was impact of vision loss on quality life.
*General*
Telemedicine and artificial intelligence to improve referral from primary to secondary care both were ranked highly and were considered likely to have an impact on equity (Supporting Information: Tables S1 and S2). There was interest in “Models for out‐of‐hours emergency eye care” and the “Diagnosis and management of eye disease by non‐ophthalmologists”—this latter question was not on the original list of questions but was added to the top five as related questions were mentioned frequently in additional comments. One new question, “Decision support tools for papilloedema referrals from primary care to secondary care” was suggested only once and therefore did not reach our criteria for presenting again in Round 2 but may reflect a recent controversial case in the United Kingdom.

### 4.1 Evaluation

The key metric from this exercise will be the publication of high‐impact high‐priority reviews. Our focus has initially been Cochrane, but the questions suggested will equally apply to non‐Cochrane systematic reviews. The list of reviews has already been useful in directing collaborations with professional organizations. For example, the Cochrane Eyes and Vision US satellite is currently working with the American Academy of Optometry to guide systematic review teams through the steps involved in developing a Cochrane systematic review using titles identified in this priority setting exercise. The priority list is also useful when responding to proposals for new reviews—for example, from stakeholders such as WHO where there is an alignment of priorities on “Models to increase uptake and access to refraction and optical services” and response to the World Health Assembly call for improving coverage of refractive error services [29].

In our protocol, we set out a plan for both short‐term and long‐term evaluation. In this report, we will not cover the long‐term evaluation as there has not been enough time for reviews to be published (Table 2).

**Table 2:** Short‐term evaluation.

Criteria	Evaluation
Did the priority setting process meet Cochrane mandatory and desirable criteria for governance, stakeholder engagement, documentation, and dissemination?	The protocol was developed based on Cochrane mandatory and desirable criteria and we have largely addressed these, for example, by establishing a Steering Group, engaging with internal and external stakeholders, publicizing the priority setting exercise, and producing a plan (protocol). Dissemination is in progress.
Was the process completed within the prespecified time frame?	We did not prespecify a time frame, but the process took longer than anticipated. It took approximately 1 year to develop and publish the protocol, part of that time relating to delays in the publication process, but we felt a published protocol was important. It took a further 2 years to conduct the searches, refine the questions, develop, conduct, and analyze the surveys and write up the process for publication.
Was the process completed without using excessive Cochrane Eyes and Vision staff time?	The process did require considerable Cochrane Eyes and Vision staff time—this partly reflected the decision to cover the whole scope of the group, which is very broad. In future, it may be better to focus on individual condition groups, for example, cataract, glaucoma, to make the process more efficient.
What did the stakeholders feel about the process? (a) What did stakeholders like about the process? (b) What did stakeholders want to improve about the process?	33 people responded to the evaluation survey. Respondents appreciated the chance to be involved and to contribute (*n* = 5) and felt that a positive part of the process was the inclusivity and collaboration in the form of engagement with a wide range of stakeholders (*n* = 11). However, two respondents felt that participation could have been wider and one noted the drop off in participation between Rounds 1 and 2 and suggested delegating dissemination to one person per country and involving relevant professional bodies in each country. Two participants mentioned that they liked getting feedback on the process. Respondents felt that the process was well‐structured and systematic (*n* = 8), covered a detailed and broad spectrum of topics in eyes and vision (*n* = 5), and overall was clear and straightforward to do (*n* = 9). One participant appreciated the opportunity to access the survey online. Three respondents felt that there could have been better explanations and definitions and a further four participants felt that there were too many options to choose from. A few respondents made general comments on the topics, recommending consideration of human resource requirements and health systems approach. One noted that the questions were broad and would need more work to make specific before doing a review. A further respondent was interested to know which questions had a universal application and which were more region specific.

## 5 DISCUSSION

We have conducted a systematic and global priority setting exercise for systematic reviews with a focus on equity. Participants were located in all parts of the world and included practitioners and patients. The resultant list of reviews will provide an important spur to organizations such as Cochrane and other evidence synthesis groups working in eyes and vision. This review adds to other priority setting exercises in Eyes and Vision, most notably the James Lind Alliance Priority Setting Partnership for Sight Loss and Vision in the United Kingdom, which is currently being updated [19]. However, in contrast to these other exercises, we focussed on questions that might usefully be answered by systematic reviews and evidence synthesis, that is, secondary research only and have included a global rather than a country‐specific focus.

The most impactful future reviews in eyes and vision are likely to be reviews focussing on the effective and equitable delivery of services, particularly for the major causes of blindness and vision impairment globally, that is, cataract and refractive error but also for eye health care for children and rehabilitation services. In particular, for cataract and refractive error questions identified arising from the Grand Challenges on Global Eye Health were ranked highly, that is, “Interventions to improve access to, and/or affordability of, cataract surgery,” “Models to increase uptake and access to refraction and optical services” and “Models for school vision screening” [20].

It was clear that respondents prioritized equity as evidenced by questions considered to have an impact on equity being prioritized. It was also suggested that disaggregated data would be a useful addition to this work. This could be done using the PROGRESS framework, which identifies relevant characteristics, that is, place of residence, race/ethnicity/culture/language, occupation, gender/sex, religion, education, socioeconomic status, social capital [30]. PROGRESS Plus includes personal characteristics such as age and disability, relationships such as being excluded from school, and time‐dependant relationships, for example, leaving hospital that might be associated with disadvantage [7]. A similar framework used in the United States is “Healthy People 2030,” which includes five domains: health care access and quality, social and community context, education access and quality, economic stability, and neighborhood and built environment [31]. Formal consideration of disaggregated data has not been common in Cochrane Eyes and Vision Reviews so far [8] but is certainly feasible as demonstrated by its use by Ramke et al. [32]. Input from a patient group highlighted that people with intellectual disabilities are an underserved group for eye care.

Reflecting concerns about the myopia epidemic and global prevalence of presbyopia, questions relating to the development and progression of refractive error and interventions for presbyopia are also priorities. The most commonly mentioned additional question related to the control of myopia progression in children. This question was not added to the priority list as it is currently being addressed by Cochrane Eyes and Vision with a network meta‐analysis in progress due to be published in 2023 [23]. Reflecting the active research area and importance to children and parents, this topic is to be maintained thereafter as a living systematic review, that is, updated regularly.

Prognosis reviews were rated highly. This is one area where Cochrane Eyes and Vision Reviews may be able to make an important impact. Methods for prognosis reviews have been developed relatively recently and Cochrane has been at the forefront of developing guidance and implementing good practice [33]. A rigorous approach to identifying and summarizing the literature on the prognosis of the major eye conditions is clearly warranted. Currently, Cochrane Eyes and Vision is supporting the development of four prognosis reviews on diabetic retinopathy [11], glaucoma (protocol in press), age‐related macular degeneration, and macular detachment (protocols in development).

### 5.1 Limitations

There are some limitations to this priority setting process. There is a tension between global reach and local understanding. Although we have tried to address global priorities, we acknowledge that local contexts may have particular economic constraints and/or different models of funding (tax vs. private), which may mean that questions relevant to local contexts may be more useful to provide evidence to change practice, particularly regarding service delivery. For example, the Fred Hollows Foundation is currently conducting a priority setting exercise for research in diabetic eye disease which aims to elicit local priorities in six different locations worldwide [34]. National health agencies, for example, NICE in the United Kingdom, typically require answers to more focused questions on the effectiveness or use of the most recent technologies, including drugs, devices, and tests. Given the global and equity perspective, the provision of evidence for approval and reimbursement, which means rapid and sometimes once‐only assessments with economic evaluation, may not be covered here.

Although overall we had a good response to our survey, with 170 people completing two rounds, with this methodology, it is difficult for us to judge whether all perspectives have been considered. Although balanced in terms of the gender of respondents, the majority of responses were from the European region and from ophthalmic practitioners, which will undoubtedly influence the choice of questions.

Due to resource constraints, we pursued an online survey format as we did not have funding to conduct face‐to‐face workshops. It was clear that it was difficult for some participants to understand the process and questions. In addition, some groups, for example, ophthalmic nurses, were underrepresented. A two‐way face‐to‐face dialog would have facilitated the involvement of individuals. However, the online approach meant we could have a global reach easily.

We limited the survey to two Delphi rounds and started the process by compiling a list of questions from scoping the literature. We also gave the opportunity to participants to suggest new questions, and these were used to add depth to the current list. We have not added questions suggested by only one participant in Round 1 to the list to be ranked in Round 2 but have presented all the suggested questions for information. Another round would have enabled ranking of all the new questions.

We sometimes found it difficult to choose between breadth and depth in review questions—for reasons of feasibility at the data collection stage, we grouped some topics. There is some unevenness in the review questions that we identified, for example, questions range from very specific, for example, “Posterior scleral reinforcement for controlling myopia progression” to very broad, for example, “Interventions for presbyopia.” For the broad questions, we envisage a separate process where review author teams focus on the PICO (participants, interventions, comparator, and outcomes) in more depth and possibly choose extensions of evidence synthesis methods such as network meta‐analysis to integrate information on many different interventions.

Finally, although we have explicitly tried to identify questions suitable for systematic reviews, there may not be sufficient high‐quality primary research (randomized controlled trials or other high‐quality evidence) to provide definite and conclusive answers to these priority systematic review questions. However, in these situations, a well‐conducted systematic review can provide a useful framework for future research.

### 5.2 Future directions

The unique aspect of Cochrane Reviews is that they aim to avoid duplication of effort by preregistration and aim to be regularly updated. We included questions for both new reviews and potential updates in this exercise. However, sometimes new questions and updates may overlap—for example, the highly ranked review on cataract “Interventions to improve access to, and/or affordability of, cataract surgery” also overlaps with a current Cochrane Review “Interventions to improve access to cataract surgical services and their impact on equity in low‐ and middle‐income countries” published in 2017 [32]. Identifying this as a priority could lead to several courses of action: the current review could be updated as a priority, or a new review on this topic could take a broader focus or consider a broader group of studies. The issue of affordability could be included or considered in a separate review. It is likely that the review questions identified in this exercise will act as a springboard rather than a directive.

The challenge will be to ensure that the priority reviews identified by this process are completed. This will require funding and collaboration. Other authors have noted the lack of impact of priority setting exercises on research, using the example of macular research in the United Kingdom [35]. Currently, our priority list is available on the Cochrane Eyes and Vision website with information about reviews that are now underway since the exercise has been completed. We are exploring ways of making this process iterative and part of a living process.

## 6 CONCLUSIONS

A global group of stakeholders prioritized questions on the effective and equitable delivery of services for eye health care. When considering the impact of systematic reviews in terms of reducing the burden of eye conditions, equity is clearly an important criterion to consider in priority‐setting exercises.

## AUTHOR CONTRIBUTIONS

**Jennifer R. Evans**: Conceptualization; data curation; formal analysis; investigation; methodology; project administration; writing—original draft. **Iris Gordon**: Conceptualization; data curation; writing—review and editing. **Augusto Azuara‐Blanco**: Conceptualization; methodology; writing—review and editing. **Michael Bowen**: Conceptualization; methodology; writing—review and editing. **Tasanee Braithwaite**: Conceptualization; methodology; writing—review and editing. **Roxanne Crosby‐Nwaobi**: Conceptualization; methodology; writing—review and editing. **Stephen Gichuhi**: Conceptualization; methodology; writing—review and editing. **Ruth E. Hogg**: Conceptualization; methodology; writing—review and editing. **Tianjing Li**: Conceptualization; methodology; writing—review and editing. **Virginia Minogue**: Conceptualization; methodology; writing—review and editing. **Roses Parker**: Conceptualization; methodology; writing—review and editing. **Fiona J. Rowe**: Conceptualization; methodology; writing—review and editing. **Anupa Shah**: Conceptualization; methodology; writing—review and editing. **Gianni Virgili**: Conceptualization; methodology; writing—review and editing. **Jacqueline Ramke**: Conceptualization; methodology; writing—review and editing. **John G. Lawrenson**: Conceptualization; data curation; investigation; methodology; writing—review and editing.

## CONFLICT OF INTEREST STATEMENT

T. J.: Cochrane Eyes and Vision US Project is funded by National Eye Institute, National Institutes of Health (grant #: UG1 EY020522). M. B.: is a full‐time employee of the College of Optometrists, and completed the work on this manuscript as part of his employment with that organization. The College is a charity, and a stakeholder in the wider work of the Cochrane Eyes and Vision Group. MB also contributes to the UKRI BBSRC/MRC funded BLAST Network (part of the UKRI's healthy aging network funding) advisory group. There is no funding attached to M. B.'s role on the advisory group for the UKRI healthy aging networks coordinating network or the BLAST network within this group, nor does any funding flow from this to the College as his employer. However, these networks have potential interests in aspects of the areas of eye health covered by this review. F. J. R.: has received grants from Fight for Sight, The Stroke Association, Haag Streit AG, and NIHR for work on rehabilitation for stroke and visual field assessment. F. J. R. has received consultancy (paid to University) from Haag Streit AG and sits on Intercollegiate Stroke Working Party and is president of Society for Research in Rehabilitation. J. R.: grants from Health Research Council, New Zealand, NZ Association of Optometrists Education & Research Fund, Blind Low Vision New Zealand, Buchanan Charitable Foundation New Zealand, Vice President, International Society of Geographic & Epidemiological Ophthalmologists (unpaid). The remaining authors declare no conflict of interest.

## ETHICS STATEMENT

This project was approved by the ethics committee of the London School of Hygiene & Tropical Medicine (22669, December 2020).

## Supporting information

Supporting information.

## Data Availability

The data that support the findings of this study are available from the corresponding author upon reasonable request.
