# Supplementary material for: Identifying important questions for Cochrane systematic reviews in Eyes and Vision: Report of a priority setting exercise
Source: Cochrane Evid Synth Methods. 2023 May 24;1(3):e12014. doi: 10.1002/cesm.12014 (PMC11795936; doi:10.1002/cesm.12014)
Supplement: Supplementary file 1 — Supporting information. [file CESM-1-e12014-s001.docx]

Contents

[Appendix 1: Cochrane Eyes and Vision Priority Setting Group Authorship 2](#_Toc134433743)

[Appendix 2: Reporting guideline for health research priority setting with stakeholders (REPRISE) 6](#_Toc134433744)

[Appendix 3: Searches and sources 12](#_Toc134433745)

[Appendix 4: Ranking of questions in Round 1 and Round 2 16](#_Toc134433746)

[Supplementary table 1: ranking of questions in Round 1 and Round 2 16](#_Toc134433747)

[Supplementary table 2: importance criteria (Round 2) 28](#_Toc134433748)

[Supplementary table 3: mapping additional questions suggested in Round 1 33](#_Toc134433749)

[Supplementary table 4: additional questions not mapped 51](#_Toc134433750)

[Supplementary table 5: additional questions out of scope 59](#_Toc134433751)

# Appendix 1: Cochrane Eyes and Vision Priority Setting Group Authorship

| **Title** | **First name** | **Initial** | **Second name** | **Country** | **Affiliation (if any)** |
| --- | --- | --- | --- | --- | --- |
| Dr | Siddharth |  | Agrawal | India | King George's Medical University, UP, India |
| Mr | Pouya |  | Alaghband | United Kingdom | Hull-York Medical School, York Teaching Hospital |
| Professor | Mehmet Numan |  | Alp | Türkiye | University of Health Sciences |
| Dr | Haroon |  | Awan | Pakistan |  |
| Professor | Balendra | P. | Singh | India | King George's Medical University, U.P., Lucknow |
| Professor | Pradeep |  | Bastola | Nepal | Chitwan Medical College |
| Dr | Nicholas | A.V. | Beare | United Kingdom | University of Liverpool |
| Professor | Mukharram | M. | Bikbov | Russia | Ufa Eye Research Institute |
| Mr | Effendy |  | Bin Hashim | Malaysia | Optometry Officer, Ministry of Health Malaysia |
| Mr | Jeewanand |  | Bist | Nepal | Dhangadi Netralaya Eye Hospital, Dhangadi |
| Dr | Richard | J. | Blanch | United Kingdom | Academic Department of Military Surgery and Trauma, Royal Centre for Defence Medicine |
| Dr | Miriam | R. | Cano | Paraguay | Catholic University of Asunción |
| Dr | Nicole | A. | Carnt | Australia | UNSW, Sydney |
| Dr | Chinomso | J. | Chinyere | Nigeria | Landmark University Medical Centre, Omu-Aran, Kwara State |
| Mr | Gerry |  | Clare | United Kingdom | Moorfields Eye Hospital NHS Foundation Trust, London |
| Professor | Qëndresë |  | Daka | Kosovo | University of Prishtina "Hasan Prishtina" |
| Dr | Joshua | R. | Ehrlich | USA | University of Michigan |
| Dr | Gamal |  | Ezz Elarab | Egypt | Magrabi Foundation |
| Dr | Helena | P. | Filipe | Portugal | West Lisbon Hospitals Center (CHLO), Hospital Egas Moniz.  Egas Moniz Center for Interdisciplinary Research (CiiEM).  Lisbon |
| Ms | Auxi | A. | Fraiz | Spain | None |
| Professor | Gus |  | Gazzard | United Kingdom | University College London |
| Dr | Ronnie |  | George | India | Medical Research Foundation, Sankara Nethralaya, Chennai |
| Dr | Rose | M. | Gilbert | United Kingdom | Moorfields Eye Hospital NHS Foundation Trust, London |
| Professor | Clare | E. | Gilbert | United Kingdom | London School of Hygiene & Tropical Medicine |
| Professor | Renata |  | Gomes | United Kingdom | BRAVO VICTOR |
| Mr | Ronnie |  | Graham | Scotland | Vision Action |
| Dr | Lisa | M. | Hamm | Aotearoa, New Zealand | University of Auckland |
| Dr | Lauren |  | Hepworth | United Kingdom | University of Liverpool |
| Mr | Luke |  | Herbert | United Kingdom | Surrey and Sussex Healthcare NHS Trust |
| Dr | Simon | J. | Hickman | United Kingdom | Royal Hallamshire Hospital, Sheffield |
| Dr | Roxane | J. | Hillier | United Kingdom | Newcastle Eye Centre, Royal Victoria Infirmary, Queen Victoria Road, Newcastle upon Tyne |
| Mr | Lewis |  | Jacobs | England | None |
| Professor | Jost | B. | Jonas | Germany | Institute of Molecular and Clinical Ophthalmology Basel |
| Mr | Obeda |  | Kailani | United Kingdom | King’s College Hospital NHS Foundation Trust, London |
| Dr | Himal |  | Kandel | Australia | The University of Sydney, Faculty of Medicine and Health, Sydney Medical School, Save Sight Institute, Sydney, NSW |
| Professor | Anthony | J. | King | United Kingdom | Nottingham University Hospital |
| Mr | Gatera Fiston |  | Kitema | Rwanda | University of Rwanda |
| Mr | Panagiotis Petros |  | Koumoutsos | Greece | General Hospital of Tripoli “Panarkadiko" |
| Dr | Michael | A. | Kwarteng | Zimbabwe | Bindura University of Science Education |
| Dr | Jonathan | C. | Lake | Brazil | Federal University of São Paulo |
| Dr | Gareth |  | Lingham | Ireland | Centre for Eye Research Ireland, Technological University Dublin |
| Dr | António | F. | Macedo | Sweden | Linnaeus University, Department of Medicine and Optometry |
| Mr | Ian |  | McCormick | United Kingdom | International Centre for Eye Health, London School of Hygiene & Tropical Medicine |
| Dr | Shaffi | Y. | Mdala | Malawi | Kamuzu University of Health Sciences, Blantyre |
| Professor | Jod |  | Mehta | Singapore | Singapore Eye Research Institute |
| Dr | Neda |  | Minakaran | United Kingdom | Kings College Hospital NHS Foundation Trust, London |
| Dr | Ashik |  | Mohamed | India | Henry Stewart Talks Ltd |
| Dr | Henrietta | I. | Monye | Nigeria | Eleta Eye Institute, Ibadan |
| Ms | Lilian |  | Nyaboga | Kenya | Kwale Eye Centre |
| Dr | Cynthia | L.A. | Ogundo | Kenya | Mbagathi Hospital |
| Dr | Oteri | E. | Okolo | Nigeria | National Eye Health Programme, Federal Ministry of Health, Abuja |
| Dr | Harry | O. | Orlans | United Kingdom | Moorfields Eye Hospital NHS Foundation Trust, London |
| Dr | Axel |  | Petzold | United Kingdom and The Netherlands | The National Hospital for Neurology and Neurosurgery, Queen Square, Moorfields Eye Hospital, London & Dutch neuro-ophthalmology Expert Center, Amsterdam UMC |
| Dr | Mapa Prabhath |  | Piyasena | Sri Lanka | Ministry of Health Sri Lanka and Centre for Public Health, Queen's University Belfast |
| Dr | Mansur | M. | Rabiu | UAE | Noor Dubai Foundation |
| Mrs | Zahra | A. | Rashid | Kenya | None |
| Dr | Alison | L. | Reynolds | Ireland | School of Veterinary Medicine and UCD Conway Institute of Biomolecular and Biomedical Research, University College Dublin |
| Mrs | Sue |  | Ricketts | United Kingdom | Nystagmus Network |
| Dr | Sare |  | Safi | Iran | Ophthalmic Epidemiology Research Center, Research Institute for Ophthalmology and Vision Science, Shahid Beheshti University of Medical Sciences, Tehran |
| Professor | Solange | R. | Salomao | Brazil | Universidade Federal de São Paulo - UNIFESP |
| Dr | Indra | P. | Sharma | Bhutan | Jigme Dorji Wangchuck National Referral Hospital, Thimphu |
| Dr | Juan Carlos |  | Silva | Colombia | Independent consultant |
| Dr | Susan | L. | Silveira | Australia | Macquarie University |
| Mr | Pushkar |  | Silwal | New Zealand | The University of Auckland |
| Dr | Rajat | M. | Srivastava | India | King George's Medical University, Lucknow |
| Professor | David | H. | Steel | United Kingdom | Bioscience Institute, Newcastle University |
| Professor | Stephen |  | Tuft | United Kingdom | Moorfields Eye Hospital NHS Foundation Trust, London |
| Ms | Juliette |  | Vila Sinclair Spence | The Netherlands | Acanthamoeba Keratitis Eye Foundation |
| Mr | Richard | P.L. | Wormald | United Kingdom | None |
| Ms | Sumrana |  | Yasmin | Pakistan | Sightsavers, UK |
| Ms | Mayinuer |  | Yusufu | China | 1. Centre for Eye Research Australia, Royal Victorian Eye and Ear Hospital, East Melbourne, Australia; 2. Department of Surgery (Ophthalmology), The University of Melbourne, Melbourne, Australia |
| Dr | Justine | H. | Zhang | United Kingdom | London School of Hygiene & Tropical Medicine |
| Dr | Emmanuel |  | Zhou | Zimbabwe | National University of Science and Technology |

# Appendix 2: Reporting guideline for health research priority setting with stakeholders (REPRISE)

| **No** | **Item** | **Descriptor and/or examples** | **Location in paper** |
| --- | --- | --- | --- |
| A | Context and scope |  |  |
| 1 | Define geographical scope | Global, regional, national, city, local area, institutional/organizational level, health service | Introduction |
| 2 | Define health area, field, focus | Disease or condition specific, interventions, healthcare delivery, health system | Introduction |
| 3 | Define the intended beneficiaries | This may include the general population or a specific population based on demographic (age, gender), clinical (disease, condition), or other characteristics who may benefit from the research | Introduction |
| 4 | Define the target audience of the priorities | Policy makers, funders, researchers, industry or others who have the potential to implement the priorities identified | Introduction |
| 5 | Identify the research area | Public health, health services research, clinical research, basic science | Introduction |
| 6 | Identify the type of research questions | Etiology, diagnosis, prevention, treatment (interventions), prognosis, health services, psychosocial, behavioral and social science, economic evaluation, implementation; this may not be pre-defined | Introduction |
| 7 | Define the time frame | Interim, short-term, long-term priorities, plans to revise and update | Results |
| B | Governance and team |  |  |
| 8 | Describe the selection and structure of the leadership and management team | Those responsible for initiating, developing, and guiding the process for priority setting, and examples of structures include; Steering Committee, Advisory Group, Technical Experts | Methods |
| 9 | Describe the characteristics of the team | Stakeholder group or role, institutional affiliations, country or region, demographics (e.g. age sex), discipline, experience, expertise | Methods |
| 10 | Describe any training or experience relevant to conducting priority setting | Consultants or advisors, members with experience or skills relevant to the conducting priority-setting e.g. qualitative methods, surveys, facilitation | Methods |
| C | Framework for priority setting |  |  |
| 11 | State the framework used (if any) | James Lind Alliance, COHRED, CHNRI, Dialogue Model, no framework (general research priority setting) | Methods |
| D | Stakeholders or participants |  |  |
| 12 | Define the inclusion criteria for stakeholders involved in priority-setting | Patients, caregivers, general community, health professionals, researchers, policy makers, non-governmental organizations, government, industry; specific groups including vulnerable and marginalized populations | Methods |
| 13 | State the strategy or method for identifying and engaging stakeholders | Partnership with organizations, social media, recruitment through hospitals | Methods |
| 14 | Indicate the number of participants and/or organizations involved | Number of individuals and organizations, include number by stakeholder group | Results |
| 15 | Describe the characteristics of stakeholders | Stakeholder group, demographic characteristics, areas of interest and expertise, discipline, affiliations | Results table 1 |
| 16 | State if reimbursement for participation was provided | Cash, vouchers, certificates, acknowledgement; what purpose e.g. travel, accommodation, honorarium | Methods |
| E | Identification and collection of research priorities |  |  |
| 17 | Describe methods for collecting initial priorities | Methods e.g. Delphi survey, surveys, nominal group technique, interviews, focus groups, meetings, workshops; prioritization e.g. voting, ranking; mode e.g. face-to-face, online; may be informed by evidence e.g. systematic reviews, reviews of guidelines/other documents, health technology assessment | Methods |
| 18 | Describe methods for collating and categorizing priorities | Taxonomy or other framework used to organize, summarise, and aggregate topics or questions | Methos |
| 19 | Describe methods and reasons for modifying (removing, adding, reframing) priorities | Based on scope, clarity, definition, duplication, other criteria | Methods |
| 20 | Describe methods for refining or translating priorities into research topics or questions | Reviewed by Steering Committee or project team | Methods |
| 21 | Describe methods for checking whether research questions or topics have been answered | Systematic reviews, evidence mapping, consultation with experts | Methods |
| 22 | Describe number of research questions or topics | Number of priorities at each stage of the process | Methods |
| F | Prioritization of research topics/questions |  |  |
| 23 | Describe methods and criteria for prioritizing research topics or questions | Methods e.g. Delphi survey, surveys, nominal group technique, interviews, focus groups, meetings, workshops; Prioritization e.g. voting, ranking; Mode e.g. face-to-face, online; Criteria e.g. need, feasibility, novelty, equity | Methods |
| 24 | State the method or threshold for excluding research topics/questions | Thresholds for ranking scores, proportions, votes; other criteria | Methods |
| G | Output |  |  |
| 25 | State the approach to formulating the research priorities | Area, topic, questions, PICO (population, intervention, comparator, outcome) | Methods |
| H | Evaluation and feedback |  |  |
| 26 | Describe how the process of prioritization was evaluated | Survey, workshop | Methods |
| 27 | Describe how priorities were fed back to stakeholders and/or to the public; and how feedback (if received) was addressed and integrated | Public meetings or workshop, newsletters, website, email, online presentations | Methods |
| I | Implementation |  |  |
| 28 | Outline the strategy or action plans for implementing priorities | Communication with target audience, via policies and funding | Discussion |
| 29 | Describe plans, strategies, or suggestions to evaluate impact | Integration in decision-making, funding allocation, review of relevant documents | Discussion |
| J | Funding and conflict of interest |  |  |
| 30 | State sources of funding | Name sources of funding for the priority-setting exercise; if relevant include the budget and/or cost | Title page |
| 31 | Declare any conflicts or competing interests | State any conflicts of interest that may be at an individual level and/or at a contextual level (e.g. political issues, controversies) that may affect the process, output or implementation. | Title page |

#

# Appendix 3: Searches and sources

**Search strategies used to search CENTRAL in the Cochrane Library**

**Cataract**

#1 MeSH descriptor: [Cataract] explode all trees

#2 MeSH descriptor: [Cataract Extraction] explode all trees

#3 #1 or #2

**Refractive error**

#1 MeSH descriptor: [Refractive Errors] explode all trees

**Diabetic retinopathy**

#1 MeSH descriptor: [Diabetic Retinopathy]

**Glaucoma**

#1 MeSH descriptor: [Glaucoma] explode all trees

**Macular degeneration**

#1 MeSH descriptor: [Macular Degeneration] explode all trees

**Amblyopia and strabismus**

#1 MeSH descriptor: [Amblyopia] this term only

#2 MeSH descriptor: [Strabismus] explode all trees

#3 #1 or #2

**Disorders of eye movement**

#1 MeSH descriptor: [Ocular Motility Disorders] explode all trees

#2 MeSH descriptor: [Eye Movements] explode all trees

#3 #1 or #2

**Infectious/ inflammatory diseases**

#1 [mh "Conjunctivitis"]

#2 [mh "Keratitis"]

#3 [mh "Uveitis"]

#4 [mh "Endophthalmitis"]

#5 #1 OR #2 OR #3 OR #4

#6 [mh blepharitis]

#7 [mh blepharospasm]

#8 [mh chalazion]

#9 [mh ectropion]

#10 [mh entropion]

#11 [mh hordeolum]

#12 [mh Blepharoptosis]

#13 #6 or #7 or #8 or #9 or #10 or #11 or #12

#14 #5 or #13

**Ocular trauma**

#1 MeSH descriptor: [Eye Injuries] explode all trees

**Ocular surface disorders**

#1 MeSH descriptor: [Dry Eye Syndromes] explode all trees

**Disorders of the eyelid and lacrimal system**

#1 [mh "Eyelid Diseases"]

#2 [mh ^"Lacrimal Apparatus Diseases"]

#3 [mh ^"Lacrimal Duct Obstruction"]

#4 [mh ^" Nasolacrimal Duct "]

#5 #1 or #2 or #3 or #4

**Eye cancer**

#1 MeSH descriptor: [Eye Neoplasms] explode all trees

**Low vision**

#1 MeSH descriptor: [Vision, Low] this term only

#2 MeSH descriptor: [Visually Impaired Persons] this term only

#3 MeSH descriptor: [Blindness] this term only

#4 #1 or #2 or #3

**Other sources**

American Academy of Ophthalmology Complementary therapy assessment marijuana in the treatment of glaucoma June 2014

American Academy of Ophthalmology Primary open-angle glaucoma Preferred Practice Pattern. 2015

American Optometric Association. Comprehensive Adult Eye and Vision Examination. 2015

American Optometric Association. Eye Care of the Patient with Diabetes Mellitus. 2019

British and Irish Orthoptic Society research Priorities

Buchan JC, Dean WH, Foster A, Burton MJ. What are the priorities for improving cataract surgical outcomes in Africa? Results of a Delphi exercise. Int Ophthalmol. 2018 Aug;38(4):1409-1414.

Chen TC, Hoguet A, Junk AK, Nouri-Mahdavi K, Radhakrishnan S, Takusagawa HL, Chen PP. Spectral-Domain OCT: Helping the Clinician Diagnose Glaucoma: A Report by the American Academy of Ophthalmology. Ophthalmology. 2018 Nov;125(11):1817-1827. doi: 10.1016/j.ophtha.2018.05.008. Epub 2018 Jul 7. Erratum in: Ophthalmology. 2019 Jun;126(6):915.

Chen PP, Lin SC, Junk AK, Radhakrishnan S, Singh K, Chen TC. The Effect of Phacoemulsification on Intraocular Pressure in Glaucoma Patients: A Report by the American Academy of Ophthalmology. Ophthalmology. 2015 Jul;122(7):1294-307. doi: 10.1016/j.ophtha.2015.03.021. Epub 2015 May 2.

Database of NIHR funded studies

Ho AC, Scott IU, Kim SJ, Brown GC, Brown MM, Ip MS, Recchia FM. Anti-vascular endothelial growth factor pharmacotherapy for diabetic macular edema: a report by the American Academy of Ophthalmology. Ophthalmology. 2012 Oct;119(10):2179-88. doi: 10.1016/j.ophtha.2012.07.058. Epub 2012 Aug 20.

Hoguet A, Chen PP, Junk AK, Mruthyunjaya P, Nouri-Mahdavi K, Radhakrishnan S, Takusagawa HL, Chen TC. The Effect of Anti-Vascular Endothelial Growth Factor Agents on Intraocular Pressure and Glaucoma: A Report by the American Academy of Ophthalmology. Ophthalmology. 2019 Apr;126(4):611-622. doi: 10.1016/j.ophtha.2018.11.019. Epub 2018 Nov 22.

Jampel HD, Singh K, Lin SC, Chen TC, Francis BA, Hodapp E, Samples JR, Smith SD. Assessment of visual function in glaucoma: a report by the American Academy of Ophthalmology. Ophthalmology. 2011 May;118(5):986-1002. doi: 10.1016/j.ophtha.2011.03.019.

Khan NA, Anwar A, Siddiqui R. Acanthamoeba Keratitis: Current Status and Urgent Research Priorities. Curr Med Chem. 2019;26(30):5711-5726. doi: 10.2174/0929867325666180510125633.

Kim SJ, Schoenberger SD, Thorne JE, Ehlers JP, Yeh S, Bakri SJ. Topical Nonsteroidal Anti-inflammatory Drugs and Cataract Surgery: A Report by the American Academy of Ophthalmology. Ophthalmology. 2015 Nov;122(11):2159-68. doi: 10.1016/j.ophtha.2015.05.014. Epub 2015 Jun 26.

Lê JT, Qureshi R, Rouse B, Twose C, Rosman L, Lindsley K, Hawkins BS, Li T. Development and content of a database of systematic reviews for eyes and vision. Eye (Lond). 2022 Apr;36(4):883-885. doi: 10.1038/s41433-021-01514-0. Epub 2021 Apr 6.

Le JT, Hutfless S, Li T, Bressler NM, Heyward J, Bittner AK, Glassman A, Dickersin K. Setting Priorities for Diabetic Retinopathy Clinical Research and Identifying Evidence Gaps. Ophthalmol Retina. 2017 Mar-Apr;1(2):94-102. doi: 10.1016/j.oret.2016.10.003

Lindsley et al. Evaluation of Clinical Questions and Patient-Important Outcomes Associated With the Treatment of Age-Related Macular Degeneration. JAMA Ophthalmol. 2018 Nov 1;136(11):1217-1225.

Ramke J, Evans JR, Habtamu E, Mwangi N, Silva JC, Swenor BK, Congdon N, Faal HB, Foster A, Friedman DS, Gichuhi S, Jonas JB, Khaw PT, Kyari F, Murthy GVS, Wang N, Wong TY, Wormald R, Yusufu M, Taylor H, Resnikoff S, West SK, Burton MJ; Grand Challenges in Global Eye Health study group. Grand Challenges in global eye health: a global prioritisation process using Delphi method. Lancet Healthy Longev. 2022 Jan;3(1):e31-e41.

Radhakrishnan S, Chen PP, Junk AK, Nouri-Mahdavi K, Chen TC. Laser Peripheral Iridotomy in Primary Angle Closure: A Report by the American Academy of Ophthalmology. Ophthalmology. 2018 Jul;125(7):1110-1120. doi: 10.1016/j.ophtha.2018.01.015. Epub 2018 Mar 2.

RoadMap: Ophthalmology - Getting It Right First Time NHS (2019)

Roadmap: Unmet Needs in Ophthalmology: A European Vision Institute-Consensus Roadmap 2019-2025

Rowe et al. The Sight Loss and Vision Priority Setting Partnership (SLV-PSP): overview and results of the research prioritisation survey process. BMJOpen 2014 DOI: 10.1136/bmjopen-2014-004905

Strategic Roadmap for Ophthalmology – Models for Future Eye Care in Singapore Executive Summary (2018)

Smith SD, Singh K, Lin SC, Chen PP, Chen TC, Francis BA, Jampel HD. Evaluation of the anterior chamber angle in glaucoma: a report by the american academy of ophthalmology. Ophthalmology. 2013 Oct;120(10):1985-97. doi: 10.1016/j.ophtha.2013.05.034. Epub 2013 Aug 23.

TFOS DEWS II report

Top 20% of CEV reviews (ranked by access and citation statistics)

Takusagawa HL, Hoguet A, Junk AK, Nouri-Mahdavi K, Radhakrishnan S, Chen TC. Swept-Source OCT for Evaluating the Lamina Cribrosa: A Report by the American Academy of Ophthalmology. Ophthalmology. 2019 Sep;126(9):1315-1323. doi: 10.1016/j.ophtha.2019.03.044. Epub 2019 Apr 4.

Waugh et al Treatments for dry age-related macular degeneration and Stargardt disease: a systematic review. NIHR. DOI 10.3310/hta22270"

Yoshizaki M, Ramke J, Zhang JH, Aghaji A, Furtado JM, Burn H, Gichuhi S, Dean WH, Congdon N, Burton MJ, Buchan J. How can we improve the quality of cataract services for all? A global scoping review. Clin Exp Ophthalmol. 2021 Jul 21. doi: 10.1111/ceo.13976. Epub ahead of print.

# Appendix 4: Ranking of questions in Round 1 and Round 2

## Supplementary table 1: ranking of questions in Round 1 and Round 2

|  | **Cataract** |  |  |
| --- | --- | --- | --- |
|  | **As ranked in Round 1**  160 participants |  | **As ranked in Round 2**  87 participants |
| 1 | Interventions to improve access to, and/or affordability of, cataract surgery | 1 | Interventions to improve access to, and/or affordability of, cataract surgery |
| 2 | Interventions to improve training of cataract surgeons and/or support staff | 2 | Interventions to improve training of cataract surgeons and/or support staff |
| 3 | Interventions for improving outcomes after complicated cataract surgery | 3 | Interventions to improve monitoring of outcomes after cataract surgery |
| 4 | Interventions to improve monitoring of outcomes after cataract surgery | 4 | Interventions to improve integration of cataract and refractive error services |
| 5 | Interventions to improve integration of cataract and refractive error services | 5 | Interventions for improving outcomes after complicated cataract surgery |
| 6 | Interventions for the implementation of biometry in lower resource settings |  |  |
| 7 | Intraocular lens design to reduce need for spectacles after cataract surgery |  |  |
| 8 | Interventions for prevention of cystoid macular oedema after cataract surgery |  |  |
| 9 | Interventions to improve case selection for cataract surgery |  |  |
| 10 | Educational interventions for people undergoing cataract surgery |  |  |

|  | **Refractive error** |  |  |
| --- | --- | --- | --- |
|  | **As ranked in Round 1**  129 participants |  | **As ranked in Round 2**  72 participants |
| 1 | Models to increase uptake and access to refraction and optical services | 1 | Models to increase uptake and access to refraction and optical services |
| 2 | Prognostic factors for the development and progression of refractive error | 2 | Practitioner training for identification of refractive error and vision problems |
| 3 | Prescribing guidelines for spectacles to improve vision outcomes | 3 | Prescribing guidelines for spectacles to improve vision outcomes |
| 4 | Interventions for presbyopia | 4 | Prognostic factors for the development and progression of refractive error |
| 5 | Practitioner training for identification of refractive error and vision problems | 5 | Interventions for presbyopia |
| 6 | Optimum frequency for eye examinations in adults |  |  |
| 7 | Corneal collagen cross linking for myopia |  |  |
| 8 | Impact of contact lenses on quality of life |  |  |
| 9 | Laser surgery for the correction of refractive error |  |  |
| 10 | Posterior scleral reinforcement for controlling myopia progression |  |  |

|  | **Diabetic retinopathy** |  |  |
| --- | --- | --- | --- |
|  | **As ranked in Round 1**  149 participants |  | **As ranked in Round 2**  68 participants |
| 1 | Artificial intelligence for screening and/or diagnosis of diabetic retinopathy | 1 | Integration of diabetic eye care services within the health service |
| 2 | Integration of diabetic eye care services within the health service | 2 | Barriers that prevent diabetic patients having regular eye checks |
| 3 | Barriers that prevent diabetic patients having regular eye checks | 3 | Interventions to increase attendance for diabetic retinopathy screening |
| 4 | Interventions to increase attendance for diabetic retinopathy screening | 4 | Artificial intelligence for screening and/or diagnosis of diabetic retinopathy |
| 5 | Non-invasive treatments for slowing down progression of diabetic retinopathy | 5 | Non-invasive treatments for slowing down progression of diabetic retinopathy |
| 6 | Changing models of care/task-shifting for delivery of diabetic eye health care |  |  |
| 7 | Personalised risk-based intervals in screening for diabetic retinopathy |  |  |
| 8 | Telemedicine for the screening and monitoring of diabetic retinopathy |  |  |
| 9 | Intravitreal therapies for high-risk proliferative diabetic retinopathy |  |  |
| 10 | Photographic methods for the diagnosis and monitoring of diabetic retinopathy |  |  |

|  | **Glaucoma** |  |  |
| --- | --- | --- | --- |
|  | **As ranked in Round 1**  115 participants |  | **As ranked in Round 2**  63 participants |
| 1 | Artificial intelligence for screening and/or diagnosis of glaucoma | 1 | Monitoring glaucoma to prevent progression, including with telemetry |
| 2 | Monitoring glaucoma to prevent progression, including with telemetry | 2 | Interventions to improve the referral pathway for glaucoma |
| 3 | Interventions to improve the referral pathway for glaucoma | 3 | Prognostic risk factors for glaucoma development and progression |
| 4 | Optical coherence tomography for the diagnosis and monitoring of glaucoma | 4 | Artificial intelligence for screening and/or diagnosis of glaucoma |
| 5 | Prognostic risk factors for glaucoma development and progression | 5 | Optical coherence tomography for the diagnosis and monitoring of glaucoma |
| 6 | Diagnostic test accuracy of different visual field tests in adult patients |  |  |
| 7 | Minimally invasive devices (MIGS) for glaucoma surgery |  |  |
| 8 | Diagnostic test accuracy of different intraocular pressure tests in adult patients |  |  |
| 9 | Medical interventions for open angle glaucoma and ocular hypertension |  |  |
| 10 | Neuroprotection for treatment of glaucoma in adults |  |  |

|  | **Macular disease** |  |  |
| --- | --- | --- | --- |
|  | **As ranked in Round 1**  130 participants |  | **As ranked in Round 2**  48 participants |
| 1 | Prognostic factors for the development and progression of AMD | 1 | Safety and efficacy of different drug delivery systems for treatment of AMD |
| 2 | Safety and efficacy of different drug delivery systems for treatment of AMD | 2 | Interventions to improve the capacity and productivity of wet AMD pathways |
| 3 | Educational interventions for wellbeing/quality of life for people with AMD | 3 | Telemedicine for the diagnosis and monitoring of AMD |
| 4 | Telemedicine for the diagnosis and monitoring of AMD | 4 | Educational interventions for wellbeing/quality of life for people with AMD |
| 5 | Interventions to improve the capacity and productivity of wet AMD pathways | 5 | Prognostic factors for the development and progression of AMD |
| 6 | Automated detection of AMD in colour fundus photography |  |  |
| 7 | Self-monitoring to prevent progression to advanced AMD |  |  |
| 8 | Optical coherence tomography for diagnosis/monitoring of macular disease |  |  |
| 9 | Vitamin D for preventing or slowing the progression of AMD |  |  |
| 10 | Clot-dissolving drugs and gas to treat sub-macular haemorrhage |  |  |

|  | **Other retinal conditions** |  |  |
| --- | --- | --- | --- |
|  | **As ranked in Round 1**  89 participants |  | **As ranked in Round 2**  37 participants |
| 1 | Interventions to reduce risk of sight loss in retinal detachments | 1 | Prognostic factors for the outcome of surgery for epiretinal membranes |
| 2 | Interventions to prevent proliferative vitreoretinopathy in retinal detachment surgery | 2 | Interventions to reduce risk of sight loss in retinal detachments |
| 3 | Prognostic factors for visual loss in inherited retinal diseases | 3 | Wide-angle imaging to detect retinal detachment in non-eyecare settings |
| 4 | Prognostic factors for the outcome of surgery for epiretinal membranes | 4 | Interventions to prevent proliferative vitreoretinopathy in retinal detachment surgery |
| 5 | Wide-angle imaging to detect retinal detachment in non-eyecare settings | 5 | Prognostic factors for visual loss in inherited retinal diseases |
| 6 | Prognostic factors for the development of retinal detachment |  |  |
| 7 | Inverted internal limiting membrane flap for treatment of large macular holes |  |  |
| 8 | Prognostic factors for the development of retinal vein occlusion |  |  |
| 9 | Interventions to treat inferior breaks following pars plana vitrectomy |  |  |
| 10 | Immediate face down posturing after retinal detachment surgery |  |  |

**Ocular surface**

|  | **As ranked in Round 1**  64 participants |  | **As ranked in Round 2**  34 participants |
| --- | --- | --- | --- |
| 1 | Interventions for dry eye | 1 | Interventions for dry eye |
| 2 | Diagnostic tests for dry eye syndrome, including biomarkers | 2 | Interventions for corneal diseases/dystrophies |
| 3 | Interventions to improve the outcomes of corneal transplantation | 3 | Interventions to improve the outcomes of corneal transplantation |
| 4 | Interventions for corneal diseases/dystrophies | 4 | Prognostic factors for development of dry eye syndrome, including biomarkers |
| 5 | Interventions for vernal keratoconjunctivitis | 5 | Diagnostic tests for dry eye syndrome, including biomarkers |
| 6 | Prognostic factors for development of dry eye syndrome, including biomarkers |  |  |
| 7 | Prognostic factors for the development and progression of keratoconus |  |  |
| 8 | Interventions for keratoconus and ectasia |  |  |
| 9 | Interventions to provide pain relief for dry eye syndrome |  |  |
| 10 | Interventions to improve outcomes after corneal injury |  |  |

**Eyelid and lacrimal system**

|  | **As ranked in Round 1**  29 participants |  | **As ranked in Round 2**  19 participants |
| --- | --- | --- | --- |
| 1 | Interventions for cancers involving the eyelid | 1 | Interventions for lacrimal duct obstruction |
| 2 | Interventions for blepharitis and meibomian gland dysfunction | 2 | Interventions for ptosis |
| 3 | Interventions for ptosis | 3 | Interventions for cancers involving the eyelid |
| 4 | Interventions for lacrimal duct obstruction | 4 | Interventions for blepharitis and meibomian gland dysfunction |
| 5 | Interventions for entropion | 5 | Interventions for entropion |
| 6 | Surgical Interventions for ectropion |  |  |
| 7 | Non-surgical interventions for acute internal hordeolum |  |  |
| 8 | Interventions for trachoma trichiasis |  |  |
| 9 | Interventions for epiphora |  |  |
| 10 | Grafts for lower eyelid retraction repair |  |  |

**Neuro-ophthalmology**

|  | **As ranked in Round 1**  98 participants |  | **As ranked in Round 2**  39 participants |
| --- | --- | --- | --- |
| 1 | Interventions for idiopathic intracranial hypertension | 1 | Interventions for optic neuritis not associated with multiple sclerosis |
| 2 | Prognostic factors for vision loss in intracranial hypertension | 2 | Prognostic factors for visual field loss in acquired brain injury |
| 3 | Screening for cerebral visual impairment | 3 | Screening for cerebral visual impairment |
| 4 | Interventions for optic neuritis not associated with multiple sclerosis | 4 | Interventions for idiopathic intracranial hypertension |
| 5 | Prognostic factors for visual field loss in acquired brain injury | 5 | Prognostic factors for vision loss in intracranial hypertension |
| 6 | Interventions for optic neuritis associated with multiple sclerosis |  |  |
| 7 | Radiological signs as a diagnostic test for intracranial hypertension |  |  |
| 8 | Interventions for eye movement disorders due to acquired brain injury |  |  |
| 9 | Interventions for ocular myasthenia gravis |  |  |
| 10 | Diagnostic algorithms for myasthenia gravis |  |  |

**Infection and inflammation**

|  | **As ranked in Round 1**  73 participants |  | **As ranked in Round 2**  29 participants |
| --- | --- | --- | --- |
| 1 | Prognostic factors for the development and progression of uveitis | 1 | Prevention of corneal infections in contact lens wearers |
| 2 | Interventions for non-infectious uveitis | 2 | Interventions for infectious uveitis |
| 3 | Prevention of corneal infections in contact lens wearers | 3 | Interventions for non-infectious uveitis |
| 4 | Interventions for the prevention and treatment of recurrent ophthalmic herpes | 4 | Prognostic factors for the development and progression of uveitis |
| 5 | Interventions for infectious uveitis | 5 | Interventions for the prevention and treatment of recurrent ophthalmic herpes |
| 6 | Systemic antibiotics for bacterial endophthalmitis |  |  |
| 7 | Interventions for the prevention and treatment of Acanthamoeba keratitis |  |  |
| 8 | Interventions for scleritis |  |  |
| 9 | Interventions for prevention of retinal damage as a result of toxoplasmosis |  |  |
| 10 | Interventions for bacterial conjunctivitis |  |  |

**Children**

|  | **As ranked in Round 1**  108 participants |  | **As ranked in Round 2**  56 participants |
| --- | --- | --- | --- |
| 1 | School vision screening programmes (methods and impact) | 1 | School vision screening programmes (methods and impact) |
| 2 | Prognostic factors for development/progression of refractive error in children | 2 | Prognostic factors for development/ progression of refractive error in children |
| 3 | Interventions for amblyopia | 3 | Screening for amblyopia during vaccination programs for rural communities |
| 4 | Screening for amblyopia during vaccination programs for rural communities | 4 | Interventions for amblyopia |
| 5 | Screening for retinoblastoma | 5 | Screening for retinoblastoma |
| 6 | Interventions to treat retinoblastoma |  |  |
| 7 | Tests for detecting strabismus in children |  |  |
| 8 | Interventions for glaucoma in children |  |  |
| 9 | Interventions for allergic conjunctivitis in children |  |  |
| 10 | Prognostic factors for the development and progression of cataracts in children |  |  |

**Rehabilitation**

|  | **As ranked in Round 1**  85 participants |  | **As ranked in Round 2**  44 participants |
| --- | --- | --- | --- |
| 1 | Models of rehabilitation including integration with eye care | 1 | Interventions to improve access and affordability of visual rehabilitation |
| 2 | Assistive technology for children and young people with low vision | 2 | Models of rehabilitation including integration with eye care |
| 3 | Assistive technology for adults with low vision | 3 | Assistive technology for adults and children with low vision* |
| 4 | Impact of vision loss on quality life | 4 | Psychological interventions to support living with visual impairment |
| 5 | Psychological interventions to support living with visual impairment | 5 | Impact of vision loss on quality life |
| 6 | Interventions to improve physical activity in people with visual impairment |  |  |
| 7 | Interventions for preventing falls in visually impaired adults |  |  |
| 8 | Timing of uptake of low vision rehabilitation |  |  |
| 9 | Vision loss as a risk factor for falls |  |  |
| 10 | Vision rehabilitation after stroke, brain injury, cerebral vision impairment |  |  |

*This question was reworded between Round 1 and Round 2

**General**

|  | **As ranked in Round 1**  86 participants |  | **As ranked in Round 2**  45 participants |
| --- | --- | --- | --- |
| 1 | Telemedicine to improve referral from primary to secondary care | 1 | Telemedicine to improve referral from primary to secondary care |
| 2 | Artificial intelligence to improve referral from primary to secondary care | 2 | Artificial intelligence to improve referral from primary to secondary care |
| 3 | Simultaneous detection of all major eye conditions | 3 | Models for out-of-hours emergency eye care |
| 4 | Models for out-of-hours emergency eye care | 4 | Simultaneous detection of all major eye conditions |
| 5 | Detection of intraocular foreign bodies | 5 | Diagnosis and management of eye disease by non-ophthalmologists * |
| 6 | Robotic-assisted surgery compared with conventional manual surgery |  |  |
| 7 | Surgical techniques for eyes damaged by injury |  |  |

* New title added

## Supplementary table 2: importance criteria (Round 2)

| **Condition group (number of respondents)** | | **Proportion responding ‘definitely or possibly yes’** | | **Proportion responding**  **‘large or moderate extent’** | |
| --- | --- | --- | --- | --- | --- |
|  |  | **Does the proposed new review (or review update) address an important uncertainty?** | **Will a new review (or review update) at this point in time resolve this uncertainty?** | **To what extent would resolving this uncertainty reduce the magnitude of vision impairment and eye health disorders?** | **To what extent would resolving this uncertainty reduce inequalities/disparities in (i.e. have an equalising effect of) the magnitude of disease or access to care for vision impairment or eye health disorders?** |
| **Cataract (n=71)** | | | | | |
| 1 | Interventions to improve access to, and/or affordability of, cataract surgery | 90% | 85% | 93% | 91% |
| 2 | Interventions to improve training of cataract surgeons and/or support staff | 89% | 88% | 91% | 80% |
| 3 | Interventions to improve monitoring of outcomes after cataract surgery | 89% | 86% | 90% | 74% |
| 4 | Interventions to improve integration of cataract and refractive error services | 86% | 70% | 80% | 79% |
| 5 | Interventions for improving outcomes after complicated cataract surgery | 87% | 76% | 65% | 61% |
| **Refractive error (n=58)** | | | | | |
| 1 | Models to increase uptake and access to refraction and optical services | 93% | 91% | 96% | 98% |
| 2 | Practitioner training for identification of refractive error and vision problems | 78% | 69% | 81% | 82% |
| 3 | Prescribing guidelines for spectacles to improve vision outcomes | 84% | 88% | 77% | 78% |
| 4 | Prognostic factors for the development and progression of refractive error | 86% | 83% | 74% | 66% |
| 5 | Interventions for presbyopia | 79% | 66% | 75% | 74% |
| **Diabetic retinopathy (n=58)** | | | | | |
| 1 | Integration of diabetic eye care services within the health service | 83% | 83% | 82% | 89% |
| 2 | Barriers that prevent diabetic patients having regular eye checks | 79% | 83% | 89% | 93% |
| 3 | Interventions to increase attendance for diabetic retinopathy screening | 86% | 85% | 93% | 93% |
| 4 | Artificial intelligence for screening and/or diagnosis of diabetic retinopathy | 86% | 84% | 79% | 79% |
| 5 | Non-invasive treatments for slowing down progression of diabetic retinopathy | 79% | 72% | 77% | 63% |
| **Glaucoma (n=51)** | | | | | |
| 1 | Monitoring glaucoma to prevent progression, including with telemetry | 90% | 82% | 92% | 90% |
| 2 | Interventions to improve the referral pathway for glaucoma | 88% | 78% | 92% | 90% |
| 3 | Prognostic risk factors for glaucoma development and progression | 88% | 82% | 92% | 86% |
| 4 | Artificial intelligence for screening and/or diagnosis of glaucoma | 88% | 80% | 82% | 78% |
| 5 | Optical coherence tomography for the diagnosis and monitoring of glaucoma | 86% | 86% | 75% | 69% |
| **Macular disease (n=35)** | | | | | |
| 1 | Safety and efficacy of different drug delivery systems for treatment of AMD | 89% | 82% | 83% | 74% |
| 2 | Interventions to improve the capacity and productivity of wet AMD pathways | 82% | 74% | 78% | 74% |
| 3 | Telemedicine for the diagnosis and monitoring of AMD | 85% | 79% | 73% | 76% |
| 4 | Educational interventions for wellbeing/quality of life for people with AMD | 85% | 84% | 62% | 87% |
| 5 | Prognostic factors for the development and progression of AMD | 85% | 79% | 75% | 71% |
| **Other retinal disease (n=25)** | | | | | |
| 1 | Prognostic factors for the outcome of surgery for epiretinal membranes | 88% | 83% | 68% | 48% |
| 2 | Interventions to reduce risk of sight loss in retinal detachments | 88% | 81% | 89% | 78% |
| 3 | Wide-angle imaging to detect retinal detachment in non-eyecare settings | 63% | 76% | 75% | 64% |
| 4 | Interventions to prevent proliferative vitreoretinopathy in retinal detachment surgery | 83% | 72% | 85% | 62% |
| 5 | Prognostic factors for visual loss in inherited retinal diseases | 92% | 81% | 59% | 56% |
| **Ocular surface disease (n=21)** | | | | | |
| 1 | Interventions for dry eye | 60% | 76% | 67% | 62% |
| 2 | Interventions for corneal diseases/dystrophies | 73% | 81% | 81% | 67% |
| 3 | Interventions to improve the outcomes of corneal transplantation | 79% | 90% | 76% | 62% |
| 4 | Prognostic factors for development of dry eye syndrome, including biomarkers | 64% | 67% | 52% | 48% |
| 5 | Diagnostic tests for dry eye syndrome, including biomarkers | 70% | 71% | 62% | 57% |
| **Eyelid (n=9)** | | | | | |
| 1 | Interventions for lacrimal duct obstruction | 56% | 56% | 44% | 56% |
| 2 | Interventions for ptosis | 67% | 67% | 56% | 56% |
| 3 | Interventions for cancers involving the eyelid | 89% | 78% | 67% | 56% |
| 4 | Interventions for blepharitis and meibomian gland dysfunction | 75% | 75% | 50% | 50% |
| 5 | Interventions for entropion | 63% | 50% | 38% | 63% |
| **Neuro-ophthalmology (n=25)** | | | | | |
| 1 | Interventions for optic neuritis not associated with multiple sclerosis | 92% | 76% | 71% | 70% |
| 2 | Prognostic factors for visual field loss in acquired brain injury | 92% | 79% | 73% | 82% |
| 3 | Screening for cerebral visual impairment | 77% | 76% | 70% | 78% |
| 4 | Interventions for idiopathic intracranial hypertension | 88% | 68% | 72% | 71% |
| 5 | Prognostic factors for vision loss in intracranial hypertension | 85% | 76% | 76% | 63% |
| **Infection and inflammation (n=19)** | | | | | |
| 1 | Prevention of corneal infections in contact lens wearers | 89% | 84% | 74% | 65% |
| 2 | Interventions for infectious uveitis | 83% | 71% | 59% | 71% |
| 3 | Interventions for non-infectious uveitis | 88% | 76% | 71% | 75% |
| 4 | Prognostic factors for the development and progression of uveitis | 83% | 71% | 65% | 63% |
| 5 | Interventions for the prevention and treatment of recurrent ophthalmic herpes | 89% | 72% | 72% | 75% |
| **Children (n=39)** | | | | | |
| 1 | School vision screening programmes (methods and impact) | 97% | 100% | 97% | 97% |
| 2 | Prognostic factors for development/ progression of refractive error in children | 92% | 89% | 92% | 90% |
| 3 | Screening for amblyopia during vaccination programs for rural communities | 84% | 84% | 66% | 68% |
| 4 | Interventions for amblyopia | 92% | 92% | 74% | 79% |
| 5 | Screening for retinoblastoma | 94% | 91% | 61% | 62% |
| **Rehabilitation (n=32)** | | | | | |
| 1 | Interventions to improve access and affordability of visual rehabilitation | 100% | 97% | 83% | 93% |
| 2 | Models of rehabilitation including integration with eye care | 100% | 100% | 87% | 97% |
| 3 | Assistive technology for adults and children with low vision | 97% | 97% | 74% | 74% |
| 4 | Psychological interventions to support living with visual impairment | 100% | 87% | 64% | 75% |
| 5 | Impact of vision loss on quality life | 76% | 82% | 69% | 79% |
| **General (n=27)** | | | | | |
| 1 | Telemedicine to improve referral from primary to secondary care | 96% | 96% | 100 | 100 |
| 2 | Artificial intelligence to improve referral from primary to secondary care | 96% | 93% | 86% | 92% |
| 3 | Models for out-of-hours emergency eye care | 81% | 84% | 44% | 60% |
| 4 | Simultaneous detection of all major eye conditions | 84% | 77% | 87% | 88% |
| 5 | Diagnosis and management of eye disease by non-ophthalmologists | 85% | 85% | 83% | 89% |

Green 90% and above; yellow 70% and above; red under 70%.

## Supplementary table 3: mapping additional questions suggested in Round 1

**Cataract**

|  | **As ranked in Round 1** | **Additional questions suggested in Round 1 mapped to questions listed in Round 1** |
| --- | --- | --- |
| 1 | Interventions to improve access to, and/or affordability of, cataract surgery | Interventions to promote awareness and behavioural shift within communities and audiences to access cataract services  Interventions to improve gender and equity in cataract services  Increase uptake of cataract surgical services by women  Triage to ensure the most in need access the services first |
| 2 | Interventions to improve training of cataract surgeons and/or support staff | Continuous quality improvement in cataract surgery  Cataract surgeons and team training in high volume cataract surgery  Effectiveness of non-ophthalmologist cataract surgeons |
| 3 | Interventions for improving outcomes after complicated cataract surgery | Planning cataract surgery for people with complex multiple eye problems  Management of patients with high-risk cataracts and role of vitreoretinal surgeon |
| 4 | Interventions to improve monitoring of outcomes after cataract surgery |  |
| 5 | Interventions to improve integration of cataract and refractive error services |  |
| 6 | Interventions for the implementation of biometry in lower resource settings | Availability of wide range of intraocular lenses where biometry is available  Revealing realistic indications for premium lenses. |
| 7 | Intraocular lens design to reduce need for spectacles after cataract surgery |  |
| 8 | Interventions for prevention of cystoid macular oedema after cataract surgery | Interventions to better recognise and treat cystoid macular oedema after cataract surgery |
| 9 | Interventions to improve case selection for cataract surgery | Triaging to ensure the most in need access the services first |
| 10 | Educational interventions for people undergoing cataract surgery | Educational information about pre-cataract and post cataract surgery in all formats to allow blind to access the information on their own and not have it read to them. |

**Refractive error**

|  | **As ranked in Round 1** | **Additional questions mapped** |
| --- | --- | --- |
| 1 | Models to increase uptake and access to refraction and optical services | Interventions to improve the affordability of refractive services  Affordability and accessibility of refractive error services in low-income settings  Quality of existing refractive error services, coverage of existing refractive error services  Aspects of effective refractive error coverage,  Models to improve equity of access to refractive error services by different population groups i.e. gender, poverty and disability.  Interventions to improve spectacle usage - perhaps including more in-depth qualitative primary research or using mixed methods.  Public-NGO-Private partnership models to strengthen spectacle supply chain  Integration of refractive error services into health systems  Early identification and improving uptake of corrective spectacle use by children in low-income settings.  Interventions to provide refractive services to children especially who are outside the school system  Access and services among LGBTQ communities |
| 2 | Prognostic factors for the development and progression of refractive error | The role of light in myopia development and myopia control  Lifestyle Interventions to reduce the risk of myopia progression  Prognostic factors for the development and progression of myopia in children |
| 3 | Prescribing guidelines for spectacles to improve vision outcomes | spectacle prescribing patterns within the GOS for children  Improving compliance to refractive correction |
| 4 | Interventions for presbyopia | Self-adjustable refractive correction for presbyopia |
| 5 | Practitioner training for identification of refractive error and vision problems | High- and low-tech solutions for replacing cycloplegic refraction given the distress they can cause to children and adults with learning disabilities (am sure other population groups affected too)  Diagnostic test accuracy of autorefractors for children in different age groups  Training of optometrists /opticians/refractionists to identify and refer other eye condition |
| 6 | Optimum frequency for eye examinations in adults |  |
| 7 | Corneal collagen cross linking for myopia |  |
| 8 | Impact of contact lenses on quality of life |  |
| 9 | Laser surgery for the correction of refractive error | The cost-effectiveness of laser surgery for the correction of refractive errors |
| 10 | Posterior scleral reinforcement for controlling myopia progression |  |

**Diabetic retinopathy**

|  | **As ranked in Round 1** | **Additional questions mapped** |
| --- | --- | --- |
| 1 | Artificial intelligence for screening and/or diagnosis of diabetic retinopathy | Artificial intelligence should be a more specific topic (like cost-effectiveness, divided by country income setting). |
| 2 | Integration of diabetic eye care services within the health service | Multidisciplinary approach to care for people with diabetes |
| 3 | Barriers that prevent diabetic patients having regular eye checks |  |
| 4 | Interventions to increase attendance for diabetic retinopathy screening | Social behaviour change communication for diabetic retinopathy  Compliance with referrals, compliance with attending full course of treatment,  Education for patients at risk of diabetic retinopathy/ importance of eye checks |
| 5 | Non-invasive treatments for slowing down progression of diabetic retinopathy | Non-VEGF treatment modalities |
| 6 | Changing models of care/task-shifting for delivery of diabetic eye health care | Access and affordability to diabetic eye care services in low resource settings.  Sustainable screening programs for diabetic retinopathy  Interventions to improve care delivery (provider and clinician focus) |
| 7 | Personalised risk-based intervals in screening for diabetic retinopathy |  |
| 8 | Telemedicine for the screening and monitoring of diabetic retinopathy |  |
| 9 | Intravitreal therapies for high-risk proliferative diabetic retinopathy | Intravitreal steroids in phakic patients with macular oedema |
| 10 | Photographic methods for the diagnosis and monitoring of diabetic retinopathy |  |

**Glaucoma**

|  | **As ranked in Round 1** | **Additional questions mapped** |
| --- | --- | --- |
| 1 | Artificial intelligence for screening and/or diagnosis of glaucoma |  |
| 2 | Monitoring glaucoma to prevent progression, including with telemetry | Additional comment: monitoring can only detect progression, not prevent it |
| 3 | Interventions to improve the referral pathway for glaucoma |  |
| 4 | Optical coherence tomography for the diagnosis and monitoring of glaucoma |  |
| 5 | Prognostic risk factors for glaucoma development and progression | Interactions between myopia and glaucoma and how myopia modifies risk  Impact of cataract extraction on glaucoma and glaucoma surgery |
| 6 | Diagnostic test accuracy of different visual field tests in adult patients | Earlier diagnosis |
| 7 | Minimally invasive devices (MIGS) for glaucoma surgery | A critical evaluation of MIGS  Non-penetrating glaucoma surgery for advanced glaucoma |
| 8 | Diagnostic test accuracy of different intraocular pressure tests in adult patients | Earlier diagnosis |
| 9 | Medical interventions for open angle glaucoma and ocular hypertension |  |
| 10 | Neuroprotection for treatment of glaucoma in adults |  |

**Macular disease**

|  | **As ranked in Round 1** | **Additional questions mapped** |
| --- | --- | --- |
| 1 | Prognostic factors for the development and progression of AMD | Nutrition  Aetiology of age-related macular degeneration including genetics |
| 2 | Safety and efficacy of different drug delivery systems for treatment of AMD |  |
| 3 | Educational interventions for wellbeing/quality of life for people with AMD |  |
| 4 | Telemedicine for the diagnosis and monitoring of AMD |  |
| 5 | Interventions to improve the capacity and productivity of wet AMD pathways | Access and affordability to AMD services and treatments in low-income settings. |
| 6 | Automated detection of AMD in colour fundus photography | Use of AI systems to predict prognosis/progression in nvAMD |
| 7 | Self-monitoring to prevent progression to advanced AMD |  |
| 8 | Optical coherence tomography for diagnosis/monitoring of macular disease |  |
| 9 | Vitamin D for preventing or slowing the progression of AMD | Nutrition |
| 10 | Clot-dissolving drugs and gas to treat sub-macular haemorrhage |  |

**Other retinal conditions**

|  | **As ranked in Round 1** | **Additional questions mapped** |
| --- | --- | --- |
| 1 | Interventions to reduce risk of sight loss in retinal detachments |  |
| 2 | Interventions to prevent proliferative vitreoretinopathy in retinal detachment surgery |  |
| 3 | Prognostic factors for visual loss in inherited retinal diseases |  |
| 4 | Prognostic factors for the outcome of surgery for epiretinal membranes |  |
| 5 | Wide-angle imaging to detect retinal detachment in non-eyecare settings |  |
| 6 | Prognostic factors for the development of retinal detachment |  |
| 7 | Inverted internal limiting membrane flap for treatment of large macular holes |  |
| 8 | Prognostic factors for the development of retinal vein occlusion |  |
| 9 | Interventions to treat inferior breaks following pars plana vitrectomy |  |
| 10 | Immediate face down posturing after retinal detachment surgery |  |

**Ocular surface**

|  | **As ranked in Round 1** | **Additional questions mapped** |
| --- | --- | --- |
| 1 | Interventions for dry eye | Important to have regular updates on the growing number of interventions for dry eye |
| 2 | Diagnostic tests for dry eye syndrome, including biomarkers | Methods to assess severity of dry eye |
| 3 | Interventions to improve the outcomes of corneal transplantation |  |
| 4 | Interventions for corneal diseases/dystrophies |  |
| 5 | Interventions for vernal keratoconjunctivitis |  |
| 6 | Prognostic factors for development of dry eye syndrome, including biomarkers |  |
| 7 | Prognostic factors for the development and progression of keratoconus |  |
| 8 | Interventions for keratoconus and ectasia |  |
| 9 | Interventions to provide pain relief for dry eye syndrome |  |
| 10 | Interventions to improve outcomes after corneal injury |  |

**Eyelid and lacrimal system**

|  | **As ranked in Round 1** | **Additional questions mapped** |
| --- | --- | --- |
| 1 | Interventions for cancers involving the eyelid |  |
| 2 | Interventions for blepharitis and meibomian gland dysfunction |  |
| 3 | Interventions for ptosis |  |
| 4 | Interventions for lacrimal duct obstruction |  |
| 5 | Interventions for entropion |  |
| 6 | Surgical Interventions for ectropion |  |
| 7 | Non-surgical interventions for acute internal hordeolum |  |
| 8 | Interventions for trachoma trichiasis |  |
| 9 | Interventions for epiphora |  |
| 10 | Grafts for lower eyelid retraction repair |  |

**Neuro-ophthalmology**

|  | **As ranked in Round 1** | **Additional questions mapped** |
| --- | --- | --- |
| 1 | Interventions for idiopathic intracranial hypertension | Interventions specifically for vision in idiopathic intracranial hypertension and perhaps more review topics regarding vision in idiopathic intracranial hypertension with versus without papilloedema |
| 2 | Prognostic factors for vision loss in intracranial hypertension | Causes of idiopathic intracranial hypertension |
| 3 | Screening for cerebral visual impairment | More effective testing protocols for cerebral visual impairment in people with intellectual disabilities |
| 4 | Interventions for optic neuritis not associated with multiple sclerosis |  |
| 5 | Prognostic factors for visual field loss in acquired brain injury |  |
| 6 | Interventions for optic neuritis associated with multiple sclerosis |  |
| 7 | Radiological signs as a diagnostic test for intracranial hypertension |  |
| 8 | Interventions for eye movement disorders due to acquired brain injury |  |
| 9 | Interventions for ocular myasthenia gravis |  |
| 10 | Diagnostic algorithms for myasthenia gravis |  |

**Infection and inflammation**

|  | **As ranked in Round 1** | **Additional questions mapped** |
| --- | --- | --- |
| 1 | Prognostic factors for the development and progression of uveitis | Influence of microbiome on uveitis |
| 2 | Interventions for non-infectious uveitis | Evidence for second line biologic agents - patients are starting to fail Adalimumab and we have nowhere to go with NHS funding  Biologic agents for the management of non-infectious uveitis.  Supported self-management/ self-treatment with topical steroids for recurrent acute anterior uveitis  Quality of life impact of local versus systemic treatments for uveitis.  Biologics in patients with uveitis undergoing cataract surgery |
| 3 | Prevention of corneal infections in contact lens wearers | Education to contact lens users of no water with contact lenses |
| 4 | Interventions for the prevention and treatment of recurrent ophthalmic herpes |  |
| 5 | Interventions for infectious uveitis | Evidence for second line biologic agents - patients are starting to fail Adalimumab and we have nowhere to go with NHS funding  Supported self-management/ self-treatment with topical steroids for recurrent acute anterior uveitis  Quality of life impact of local versus systemic treatments for uveitis.  Biologics in patients with uveitis undergoing cataract surgery |
| 6 | Systemic antibiotics for bacterial endophthalmitis |  |
| 7 | Interventions for the prevention and treatment of Acanthamoeba keratitis |  |
| 8 | Interventions for scleritis |  |
| 9 | Interventions for prevention of retinal damage as a result of toxoplasmosis |  |
| 10 | Interventions for bacterial conjunctivitis |  |

**Children**

|  | **As ranked in Round 1** | **Additional questions mapped** |
| --- | --- | --- |
| 1 | School vision screening programmes (methods and impact) | Integrated school health programming  Compliance with spectacles/treatment  Does school vision screening result in children accessing the glasses they need |
| 2 | Prognostic factors for development/progression of refractive error in children | Prognostic factors for the development and progression of myopia in children |
| 3 | Interventions for amblyopia |  |
| 4 | Screening for amblyopia during vaccination programs for rural communities |  |
| 5 | Screening for retinoblastoma |  |
| 6 | Interventions to treat retinoblastoma |  |
| 7 | Tests for detecting strabismus in children |  |
| 8 | Interventions for glaucoma in children |  |
| 9 | Interventions for allergic conjunctivitis in children |  |
| 10 | Prognostic factors for the development and progression of cataracts in children |  |

**Rehabilitation**

|  | **As ranked in Round 1** | **Additional questions mapped** |
| --- | --- | --- |
| 1 | Models of rehabilitation including integration with eye care | Access to low vision services  interventions to improve access and affordability of visual rehabilitation  Access and affordability to rehabilitation services in low resource settings  Integration of eye care and rehabilitation sectors  Integrating low vision care and refractive error services |
| 2 | Assistive technology for children and young people with low vision | Interventions to improve the accessibility and affordability (to individuals and national governments) of assistive technology interventions  Impact of assistive technology on quality of life of people with low vision  Use of augmented reality in rehabilitation |
| 3 | Assistive technology for adults with low vision |  |
| 4 | Impact of vision loss on quality life |  |
| 5 | Psychological interventions to support living with visual impairment |  |
| 6 | Interventions to improve physical activity in people with visual impairment |  |
| 7 | Interventions for preventing falls in visually impaired adults |  |
| 8 | Timing of uptake of low vision rehabilitation |  |
| 9 | Vision loss as a risk factor for falls |  |
| 10 | Vision rehabilitation after stroke, brain injury, cerebral vision impairment |  |

**General**

|  | **As ranked in Round 1** | **Additional questions mapped** |
| --- | --- | --- |
| 1 | Telemedicine to improve referral from primary to secondary care | Models to reduce non-attendance at eye clinics |
| 2 | Artificial intelligence to improve referral from primary to secondary care |  |
| 3 | Simultaneous detection of all major eye conditions |  |
| 4 | Models for out-of-hours emergency eye care |  |
| 5 | Detection of intraocular foreign bodies | Management of ocular trauma |
| 6 | Robotic-assisted surgery compared with conventional manual surgery |  |
| 7 | Surgical techniques for eyes damaged by injury |  |
| New question added to Round 2 | Diagnosis and management of eye disease by non-ophthalmologists | Non-ophthalmologist cataract surgeons’ effectiveness  Glaucoma diagnosis and management by non-medical professionals  Training of optometrists /opticians/refractionists to identify and refer other eye conditions  Nurse led primary care services  Shared care of new and follow up patients between hospital eye service and community optometry |

## Supplementary table 4: additional questions not mapped

| **Cataract** |  |  |  |  |
| --- | --- | --- | --- | --- |
| **Prognosis/aetiology** |  | **Identification** | **Treatment** | **Health service delivery, including training** |
|  |  |  | Interventions to treat unilateral childhood cataract (including surgical interventions, prescribing, patching etc) |  |
|  |  |  | Efficacy of multifocal IOL in children undergoing cataract surgery |  |
|  |  |  | Impact of cataract extraction on glaucoma and glaucoma surgery |  |
|  |  |  | Preoperative investigations, such as haemoglobin, blood sugar (Cochrane Review (no plans to update) |  |
|  |  |  | Interventions for the implementation of management of posterior capsule opacification in lower resource settings |  |
|  |  |  | Pre-operative interventions that include patient preparation, counselling, consent, check iops among others |  |
|  |  |  | Treatment of post-operative infections following cataract surgery |  |
|  |  |  | Biologics in patients with uveitis undergoing cataract surgery |  |
| **Refractive error** |  |  |  |  |
| **Prognosis/aetiology** |  | **Identification** | **Treatment** | **Health service delivery, including training** |
| Stigma and wearing glasses |  | Strategies for identifying hyperopia in children and young adults | Compliance with spectacle wear and myopia management in low- and middle-income countries | Models for screening, diagnosing and management of keratoconus |
|  |  | Diagnostic test accuracy of autorefractors for children in different age groups | Strategies for managing hyperopia in children and young adults |  |
|  |  |  | 8 additional questions relating to intervention to halt or slow down myopia progression (Current Cochrane living systematic review in progress) |  |
| **Diabetic retinopathy** |  |  |  |  |
| **Prognosis/aetiology** |  | **Identification** | **Treatment** | **Health service delivery, including training** |
|  |  | Sustainable screening programs for diabetic retinopathy | Intravitreal steroids in phakic patients with macular oedema | Interventions to improve the teaching of retinal laser for diabetic retinopathy including macular oedema |
|  |  |  | Timing of vitrectomy in management of proliferative diabetic retinopathy |  |
|  |  |  | Laser versus anti-VEGF for proliferation retinopathy - long term outcomes by intention to treat (Cochrane Review update (in press)) |  |
|  |  |  | Comparison of non-FDA approved anti-VEGF drugs with approved drugs in terms of efficacy and safety in eyes with diabetic macular edema (Cochrane Review update (in press)) |  |
|  |  |  | Diabetic retinopathy in diabetics on biosimilar Insulin |  |
| **Glaucoma** |  |  |  |  |
| **Prognosis/aetiology** |  | **Identification** | **Treatment** | **Health service delivery, including training** |
|  |  |  | Sustained release treatments versus topical treatments | Access and affordability to glaucoma services & treatments in low-income settings |
|  |  |  | Development of a one-time surgical technique for open-angle glaucoma, as safe and successful as YAG-iridotomy for angle-closure glaucoma | District-based intervention models for prevention and control of glaucoma |
|  |  |  | Optimal laser modality for primary treatment of open angle glaucoma (Cochrane Review update published recently) |  |
|  |  |  | Effectiveness of selective laser trabeculoplasty in the Treatment of open angle glaucoma (Cochrane Review update published recently) |  |
| **Macular disease** |  |  |  |  |
| **Prognosis/aetiology** |  | **Identification** | **Treatment** | **Health service delivery, including training** |
|  |  | How to increase public awareness of macular hole symptoms to speed up asking for help and thereby getting a diagnosis and treatment | Other drugs in Stargardts disease |  |
|  |  |  | Other options for dry AMD |  |
|  |  |  | Accurate phase 4 information for Anti-VEGF drugs (rate of non-responders, those that become refractory) |  |
|  |  |  | Anti-VEGF for AMD (Cochrane Review updated/recently published) |  |
|  |  |  | Interventions to treatment of Central Serous Chorioretinopathy (Cochrane Review update in progress) |  |
|  |  |  | Metformin to reduce progression of AMD |  |
| . |  |  | Safety profile of brolicizumab compared to other anti-VEGF agents (Cochrane Review updated/recently published) |  |
| . |  |  | Magnifying intraocular lens implants for AMD (Cochrane Review available) |  |
|  |  |  | Interventions for vitelliform dystrophy |  |
|  |  |  | Silicone oil tamponade for failed macular hole surgery |  |
|  |  |  | Surgical treatment of AMD |  |
|  |  |  | Comparison of non-fda approved antiVEGF drugs with approved drugs in terms of efficacy and safety in eyes with AMD (Cochrane Review updated/recently published) |  |
|  |  |  | Low vision management of macular disease for improved quality of life |  |
| **Retinal disease** |  |  |  |  |
| **Prognosis/aetiology** |  | **Identification** | **Treatment** | **Health service delivery, including training** |
| Likelihood of retinal detachment after successful surgery |  | Screening for Sickle Cell Retinopathy | Interventions to treat Coats' Disease |  |
| Psychological impact of genetic testing for inherited retinal diseases |  | Screening for hydroxychloroquine retinopathy | Vitrectomy in the management of uveitis and endophthalmitis |  |
| **Ocular surface disease** |  |  |  |  |
| **Prognosis/aetiology** |  | **Identification** | **Treatment** | **Health service delivery, including training** |
| Prediction and prevention and allergic eye disease and vernal keratoconjunctivitis |  |  | Corneal infection is missing; there is too much concentration on dry eye and corneal abrasions |  |
|  |  |  | Contact lens related discomfort |  |
|  |  |  | Fungal Keratitis in Africa |  |
| **Eyelid and lacrimal system** |  |  |  |  |
| **Prognosis/aetiology** |  | **Identification** | **Treatment** | **Health service delivery, including training** |
| Natural history of patients reporting watery eyes |  |  | Orbito-ocular tumours | Interventions to assure quality of surgery to correct eyelid deformity secondary to repeated bacterial conjunctival infections. |
| **Neuro-ophthalmology** |  |  |  |  |
| **Prognosis/aetiology** |  | **Identification** | **Treatment** | **Health service delivery, including training** |
| Causes and prognosis for infantile nystagmus |  | Screening for intracranial hypertension | Interventions for AION to protect the fellow eye |  |
| Causes of idiopathic intracranial hypertension |  | What tests and monitoring regimes are optimal for ophthalmic surveillance in patients with suprasellar tumours? | Other causes of significant visual loss e.g. Giant cell arteritis |  |
| Ocular biomarkers for prognosis in mild traumatic brain injury |  | Non-invasive diagnosis of idiopathic intracranial hypertension | Interventions for infantile nystagmus |  |
| Ocular biomarkers for prognosis in neurodegenerative diseases |  |  | Management of sight threatening tumours e.g. pituitary, meningiomas |  |
| Onset of convergent squint associated with smart phone use |  |  | Gestational management in idiopathic intracranial hypertension |  |
| Pathogenesis of vision loss in idiopathic intracranial hypertension |  |  | Botox in the management of strabismus |  |
| **Infection** |  |  |  |  |
| **Prognosis/aetiology** |  | **Identification** | **Treatment** | **Health service delivery, including training** |
| The risk of anti microbial resistance and ocular/conjunctival infections (Non-Cochrane Review in progress) |  | Diagnosis of uveitis associated with positive TB test (IGRA or Mantoux) | Development of vaccines for bacterial infections affecting millions of people |  |
| Measurement of inflammatory activity and outcomes in uveitis. |  |  | Anti-tuberculous treatment combination and duration for ocular tuberculosis |  |
| Intraocular injury and inflammation and infection |  |  | Biologic agents for the management of non-infectious uveitis. |  |
| **Children** |  |  |  |  |
| **Prognosis/aetiology** |  | **Identification** | **Treatment** | **Health service delivery, including training** |
| Prognostic factors for late-onset convergent squint (esotropia) |  | Screening for cerebral visual impairment | Inherited retinal disease (congenital) | Accessibility and affordability to eye care services for children in low resource settings |
|  |  | Affordable screening for pre-school children in LMICs | How to improve adherence to glasses for children with intellectual disabilities | Improving training and prioritization of pediatric eye health |
|  |  |  | Patient education (guardians) for cerebrovisual impairment | Practitioners skill in assessing refractive error in children and guidelines to prescribe spectacles in children |
|  |  |  | Interventions for myopia in children (Current Cochrane living systematic review in press) |  |
|  |  |  | Interventions to close the loop of care for those identified with vision loss |  |
|  |  |  | Interventions among children not attending schools |  |
|  |  |  | Eye health promotion interventions tackling stigma and stereotyping |  |
| **Rehabilitation** |  |  |  |  |
| **Prognosis/aetiology** |  | **Identification** | **Treatment** | **Health service delivery, including training** |
| Early interventions for infants/young children with profound vision impairment of early onset |  | There is a lack of a clinically validated functional vision assessment tool that can be used with these populations but would be incredibly helpful to have | Cost effectiveness of vision rehabilitation interventions |  |
| Employment Barriers for the people with low vision |  |  | Rehabilitation for children with Cerebral visual impairment, management and pathways for suspected cerebral visual impairment |  |
|  |  |  | All of the above tend to focus on the cognitively able population, it would be helpful to see research that addresses the needs of those less cognitively able (dementia, intellectual disabilities) to see what interventions in all the highlighted cases may work |  |
| **General** |  |  |  |  |
| **Prognosis/aetiology** |  | **Identification** | **Treatment** | **Health service delivery, including training** |
|  |  | Decision support tools for papilloedema referrals from primary care to secondary case |  | Models of funding for eye care services, e.g. privately funded vs government funded services |
|  |  |  |  | Surgical training in ophthalmology, objective measure of manual dexterity for trainees considering ocular surgical specialty |

## Supplementary table 5: additional questions out of scope

| **Cataract** |
| --- |
| The development of a comprehensive eye health service addressing the leading causes of VI including blindness |
| The magnitude of eye health problem is not known due to lack fresh evidence. So need to prioritize RAAB/national surveys |
| Review of NHS National Cataract Service Specification implementation |
| **Refractive error** |
| Impact of refractive care on quality of life |
| Impact of refractive care on employment and productivity |
| Prevalence of refractive error in the learning disabled and autistic populations |
| Aspects of effective refractive error coverage |
| Quality of existing refractive error services, coverage of existing refractive error services |
| Refractive error atlas (like the Diabetes Atlas from IDF) |
| Social, economic impact of uncorrected refractive error among adults |
| Educational impact of uncorrected refractive error |
| **Diabetic retinopathy** |
| **Glaucoma** |
| Coverage and quality of glaucoma services around the world |
| Impact of Glaucoma treatment on Health related quality of life |
| **Macular disease** |
| Coverage and quality of macular disease services |
| Impact of AMD treatment on Health related quality of life |
| **Retinal disease** |
| Ocular Adverse Effects of drugs for non-ocular conditions |
| **Ocular surface disease** |
| **Eyelid and lacrimal system** |
| **Neuro-ophthalmology** |
| gestational management in IIH |
| 7 additional questions relating to management of headache or cognitive impairment in Idiopathic Intracranial Hypertension |
| **Infection** |
| Quality of life impact of local versus systemic treatments for uveitis |
| **Children** |
| Prevalence of childhood vision impairment |
| Guidelines for managing RE, strabismus and amblyopia in LMICs and low resource settings |
| Visual processing in autistic children, |
| 8 additional questions on retinopathy of prematurity detection and treatment |
| **Rehabilitation** |
| **General** |
| Prevalence of sight loss conditions in people with intellectual disabilities (overall prevalence of sight problems known but specific ocular conditions such as glaucoma, AMD not researched) |
| spectacle prescribing patterns within the GOS for children |
| Prevalence of sight loss conditions in people with intellectual disabilities (overall prevalence of sight problems known but specific ocular conditions such as glaucoma, AMD not researched) |
| Methods which can improve on visual acuity as a measure of visual function and disability |
| A still very under researched area is autism and sight loss - in particular what is the intersection between 'sensory processing difficulties' seen in autism and cerebral visual impairment |
